# *Bis*-Indolyl Benzenoids, Hydroxypyrrolidine Derivatives and Other Constituents from Cultures of the Marine Sponge-Associated Fungus *Aspergillus candidus* KUFA0062

**DOI:** 10.3390/md16040119

**Published:** 2018-04-06

**Authors:** Suradet Buttachon, Alice A. Ramos, Ângela Inácio, Tida Dethoup, Luís Gales, Michael Lee, Paulo M. Costa, Artur M. S. Silva, Nazim Sekeroglu, Eduardo Rocha, Madalena M. M. Pinto, José A. Pereira, Anake Kijjoa

**Affiliations:** 1 ICBAS-Instituto de Ciências Biomédicas Abel Salazar, Rua de Jorge Viterbo Ferreira, 228, 4050-313 Porto, Portugal; lgales@ibmc.up.pt (L.G.); pmcosta@icbas.up.pt (P.M.C.); erocha@icbas.up.pt (E.R.); 2 Interdisciplinary Centre of Marine and Environmental Research (CIIMAR), Terminal de Cruzeiros do Porto de Lexões, Av. General Norton de Matos s/n, 4450-208 Matosinhos, Portugal; nokrari_209@hotmail.com (S.B.); ramosalic@gmail.com (A.A.R.); angelainacio@gmail.com (Â.I.); 3 Department of Plant Pathology, Faculty of Agriculture, Kasetsart University, Bangkok 10240, Thailand; tdethoup@yahoo.com; 4 Instituto de Biologia Molecular e Celular (i3S-IBMC), Universidade do Porto, Rua de Jorge Viterbo Ferreira, 228, 4050-313 Porto, Portugal; 5 Department of Chemistry, University of Leicester, University Road, Leicester LE 7 RH, UK; ml34@leicester.ac.uk; 6 Departamento de Química & QOPNA, Universidade de Aveiro, 3810-193 Aveiro, Portugal; artur.silva@ua.pt; 7 Medicinal and Aromatic Plant Programme, Plant and Animal Sciences Department, Vocational School, Kilis 7 Aralık University, 79000 Kilis, Turkey; nsekeroglu@gmail.com; 8 Laboratório de Química Orgânica, Departamento de Ciências Químicas, Faculdade de Farmácia, Universidade do Porto, Rua de Jorge Viterbo Ferreira, 228, 4050-3 13 Porto, Portugal; madalena@ff.up.pt

**Keywords:** *Aspergillus candidus*, Aspergillaceae, sponge-associated fungus, *bis*-indolyl benzenoids, hydroxypyrrolidine, antibacterial activity, cytotoxicity

## Abstract

A previously unreported *bis*-indolyl benzenoid, candidusin D (**2e**) and a new hydroxypyrrolidine alkaloid, preussin C (**5b**) were isolated together with fourteen previously described compounds: palmitic acid, clionasterol, ergosterol 5,8-endoperoxides, chrysophanic acid (**1a**), emodin (**1b**), six *bis*-indolyl benzenoids including asterriquinol D dimethyl ether (**2a**), petromurin C (**2b**), kumbicin B (**2c**), kumbicin A (**2d**), 2″-oxoasterriquinol D methyl ether (**3**), kumbicin D (**4**), the hydroxypyrrolidine alkaloid preussin (**5a**), (3*S*, 6*S*)-3,6-dibenzylpiperazine-2,5-dione (**6**) and 4-(acetylamino) benzoic acid (**7**), from the cultures of the marine sponge-associated fungus *Aspergillus candidus* KUFA 0062. Compounds **1a**, **2a–e**, **3**, **4**, **5a–b**, and **6** were tested for their antibacterial activity against Gram-positive and Gram-negative reference and multidrug-resistant strains isolated from the environment. Only **5a** exhibited an inhibitory effect against *S. aureus* ATCC 29213 and *E. faecalis* ATCC29212 as well as both methicillin-resistant *S. aureus* (MRSA) and vancomycin-resistant enterococci (VRE) strains. Both **1a** and **5a** also reduced significant biofilm formation in *E. coli* ATCC 25922. Moreover, **2b** and **5a** revealed a synergistic effect with oxacillin against MRSA *S. aureus* 66/1 while **5a** exhibited a strong synergistic effect with the antibiotic colistin against *E. coli* 1410/1. Compound **1a**, **2a–e**, **3**, **4**, **5a–b**, and **6** were also tested, together with the crude extract, for cytotoxic effect against eight cancer cell lines: HepG2, HT29, HCT116, A549, A 375, MCF-7, U-251, and T98G. Except for **1a**, **2a**, **2d**, **4,** and **6**, all the compounds showed cytotoxicity against all the cancer cell lines tested.

## 1. Introduction

*Aspergillus candidus* (Family Aspergillaceae) is a member of *Aspergillus* section *Candidi* [1]. This species frequently contaminates stored food and feeding stuff [2], and is one of the most frequently encountered mold in cereal grains and flour [3]. It also occurs in soil, usually on seeds or in the rhizosphere, and also in milk [4]. Strains of *A. candidus* produce a variety of secondary metabolites including chlorflavonin, a chlorine containing flavone antifungal antibiotic [5], *p*-terphenyl derivatives such as terphenyllin [6], deoxyterphenyllin [7], 3-hydroxyterphenyllin [8], candidusins A and B [9], immunosuppressant terprenins [10]. On the other hand, there are only a few reports on the chemical investigation of marine-derived *A. candidus*. Wei et al. [11] reported the isolation of cytotoxic prenylterphyllin, 4″-deoxyprenylterphyllin, 4″-deoxyisoterprenin and 4″-deoxyterprenin from *A. candidus* IF10, isolated from marine sediment. Recently, Wang et al. [12] described the isolation of spiculisporic acid derivatives including the new compounds, spiculisporic acids F and G. 

In our pursuit for antibiotic and anticancer compounds from marine-derived fungi from the tropical sea, we have investigated secondary metabolites from cultures of *A. candidus* KUFA 0062, which were isolated from the marine sponge *Epipolasis* sp., collected from the coral reef at the Similan Island National Park in Phang-Nga province, Southern Thailand. 

**Figure 1:**
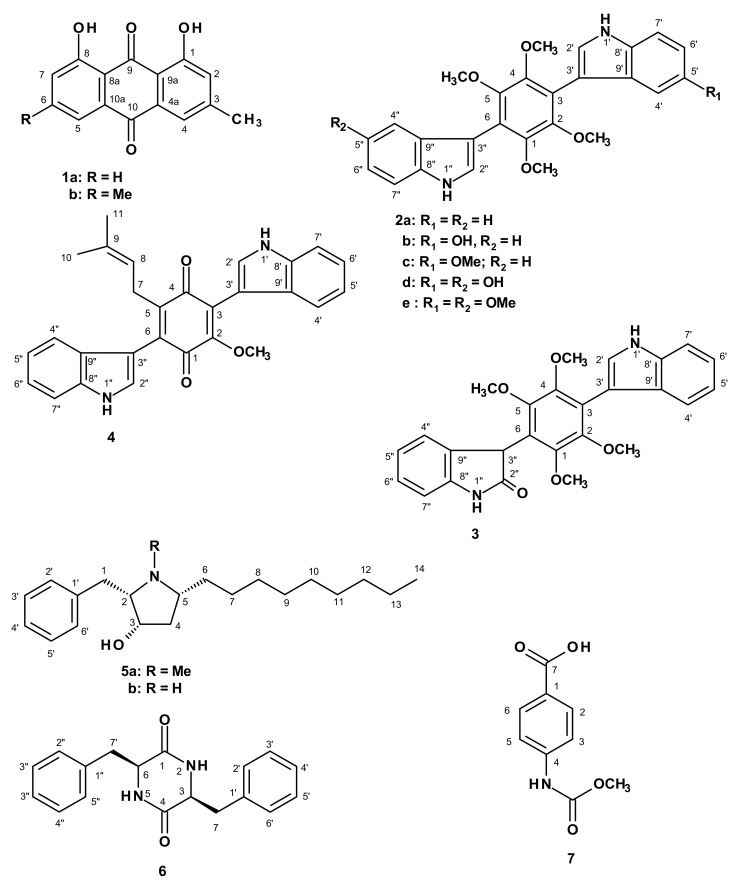
Structures of some secondary metabolites isolated from the cultures of the marine sponge-associated fungus *A. candidus* KUFA 0062.

Chromatographic fractionation and further purification of the crude ethyl acetate extract of the cultures of *A. candidus* KUFA 0062, furnished two previously undescribed compounds named candidusin D (**2e**) and preussin C (**5b**), as well as the previously reported chrysophanic acid (**1a**) [13], emodin (**1b**) [14], six *bis*-indolyl benzenoids including asterriquinol D dimethyl ether (**2a**) [15], petromurin C (**2b**) [16], kumbicin B (**2c**) [15], kumbicin A (**2d**) [15], 2″-oxoasterriquinol D methyl ether (**3**) [17], kumbicin D (**4**) [15], the hydroxypyrrolidine alkaloid preussin (**5a**) [18,19,20], (3*S*, 6*S*)-3,6-dibenzylpiperazine-2,5-dione (**6**) [21], and 4-(acetylamino) benzoic acid (**7**) [22] (Figure 1). Additionally, the common fungal metabolites, i.e., palmitic acid, clionasterol [23], and ergosterol 5,8-endoperoxides [14] were also isolated (Supplementary Material, Figure S1).

Compounds **1a**, **2a–e**, **3**, **4**, **5a–b**, and **6** were tested for their antibacterial activity against four reference bacterial strains consisting of two Gram-positive (*Staphylococcus aureus* ATCC 29213 and *Enterococcus faecalis* ATCC 29212) and two Gram-negative bacteria (*Escherichia coli* ATCC 25922 and *Pseudomonas aeruginosa* ATCC 27853), three multidrug-resistant isolates from the environment, MRSA *S. aureus* 66/1, VRE *E. faecalis* B3/101, a colistin-resistant *E. coli* 1418/1, and a clinical isolate ESBL *E. coli* SA/2. The isolated compounds were also investigated for their capacity to inhibit biofilm formation in the four reference strains as well as for their potential synergism with the clinically used antibiotics against multidrug-resistant isolates from the environment. Moreover, these compounds were also evaluated for their cytotoxic effect against eight cancer cell lines, i.e., Hep G2 (human hepatocellular carcinoma), HT29 (human colorectal adenocarcinoma), HCT116 (human colorectal carcinoma), A549 (human lung carcinoma), A375 (human malignant melanoma), MCF7 (human mammary gland adenocarcinoma), U251 (human glioblastoma multiforme), and T98G (human glioblastoma astrocytoma), by MTT assay.

## 2. Results and Discussion

The structures of palmitic acid, clionasterol [22], ergosterol 5,8-endoperoxides [14], chrysophanic acid (**1a**) [13], emodin (**1b**) [14], asterriquinol D dimethyl ether (**2a**) [15], petromurin C (**2b**) [16], kumbicin B (**2c**) [15], kumbicin A (**2d**) [15], 2″-oxoasterriquinol D methyl ether (**3**) [17], kumbicin D (**4**) [15], preussin (**5a**) [18,19,20], (3*S*, 6*S*)-3,6-dibenzylpiperazine-2,5-dione (**6**) [21] and 4-(acetylamino) benzoic acid (**7**) [22] were elucidated by analysis of their 1D and 2D NMR spectra as well as HRMS data, and also by comparison of their spectral data to those reported in the literature (Supplementary Materials, Figures S2–S17, S20–S25, S32–S35 and Tables S1–S4).

Compound **2e** was isolated as a white solid (m.p. 299–300 °C), and its molecular formula C_28_H_28_N_2_O_6_ was established based on its (+)-HRESIMS *m*/*z* 489.2030 [M + H]^+^, (calculated 489.2026 for C_28_H_29_N_2_O_6_), indicating sixteen degrees of unsaturation. The IR spectrum showed absorption bands for amine (3346 cm^−1^), aromatic (1625, 1579 cm^−1^), and ether (1291 cm^−1^) groups. The general features of the ^1^H and ^13^C NMR spectra of **2e** (Table 1, Supplementary Materials, Figures S18 and S19) resembled those of kumbicin A (**2d**), petromurin D (**2b**), and two *bis*-indolyl benzenoids isolated from the soil fungus *Aspergillus kumbius* [15]. The ^13^C NMR spectrum (Table 1) exhibited twelve carbon signals which, in combination with DEPTs and HSQC spectra, can be categorized in six quaternary sp^2^ (δ_C_ 153.1, 147.6, 131.0, 127.4, 122.0, 106.7), four methine sp^2^ (δ_C_ 126.0, 111.9, 110.9, 102.1), and two methoxyl (δ_C_ 60.3, 55.3) carbon signals. As the number of the carbon signals is less than the number of carbon atoms found in HRMS, the molecule must be symmetrical. The presence of the 3,5-disubstituted indole moiety with the methoxyl group on C-5 was supported by the presence of an amine proton at δ_H_ 11.15 (d, *J* = 2.2 Hz), a doublet at δ_H_ 7.43 (*J* = 2.5 Hz, H-2; δ_C_ 126.0), and the proton signals of the 1,2,3 substituted benzene ring at δ_H_ 6.78, dd (*J* = 8.8, 2.5 Hz, H-6; δ_C_ 110.9), 6.88, d (*J* = 2.5 Hz, H-4; δ_C_ 102.1), 7.33, dd (*J* = 8.8, H-7; δ_C_ 111.9) as well as a singlet of OMe at δ_H_ 3.72 (δ_C_ 55.3). That another portion of the molecule was 2,3,5,6-tetramethoxy-1,6-disubstituted benzene ring was supported by the presence of the carbon signals at δ_H_ 122.0 (C-1 and C-6) and δ_C_ 147.6 (C-2, C-3, C-5, C-6) as well as four chemically and magnetically equivalent methoxyl groups at δ_H_ 3.46 (δ_C_ 60.3). Putting together the HRMS and NMR data, the structure of **2e** was established as 5′-methoxykumbicin B. However, instead of having one methoxyl group and one hydroxyl group on C-5′ and C-5″ of the indole ring system like petromurin D, the substituents on both C-5′ and C-5″ are methoxyl groups in **2e**. To the best of our knowledge, **2e** is a new analogue of *bis*-indolyl benzenoids, which was named candidusin D.

**Figure 2:**
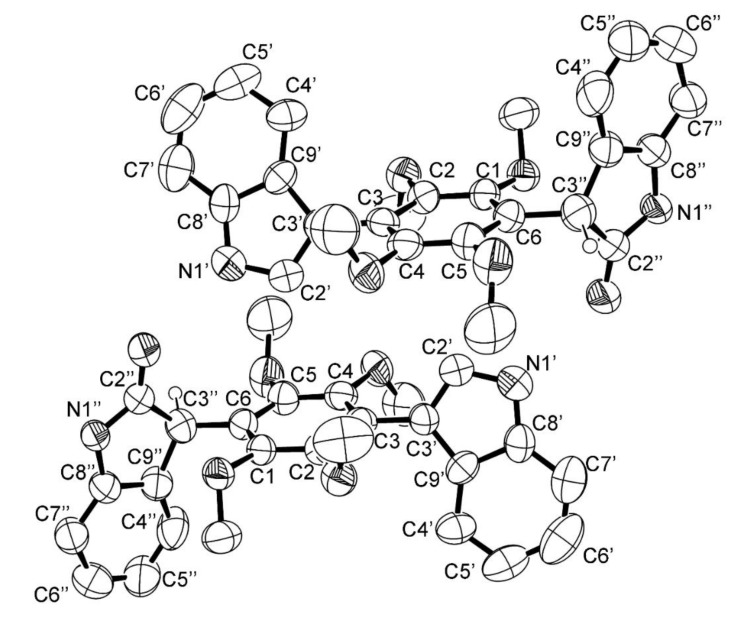
ORTEP (Oak Ridge Thermal-Ellipsoid Plot Program) view of **3**.

Compound **3** was isolated as white crystals (m.p. 290–292 °C) with [α${]}_{D}^{25}$ − 115.4 (*c* 0.04, MeOH). Analysis of the (+)-HRESIMS, ^1^H, ^13^C NMR, COSY, HSQC, HMBC data of **3** (Table S3, Supplementary Materials, Figures S20 and S21), revealed that this compound has the same planar structure as that 2″-oxoasterriquinol D methyl ether, an oxidized *bis*-indolyl benzenoid previously isolated from extracts of the sclerotia of *Aspergillus sclerotiorum* (NRRL 5167) [17]. Although Whyte et al. [17] have found that 2″-oxoasterriquinol D methyl ether was optically active, with [α${]}_{D}^{25}$ − 13.9 (*c* 0.001 g/mL, MeOH), they did not determine the absolute configuration of the stereogenic carbon (C-3″) of the oxidized indole ring system of this compound. In order to fully elucidate the structure of **3**, we have attempted to obtain suitable crystal for X-ray analysis. The results obtained, as seen in the ORTEP (Oak Ridge Thermal-Ellipsoid Plot Program) view in Figure 2, revealed that although **3** was levorotatory, it was not optically pure since both enantiomers were present in the crystal.

The (+)-HRESIMS, ^1^H, ^13^C NMR (Table 2), COSY, HSQC, and HMBC (Supplementary Materials, Figures S24 and S25) data of **5a** revealed that its planar structure is equal to that of the antifungal compound L-657, 398, first isolated from the fermentation of *Aspergillus ochraceus* ATCC 22947 [18]. However, the authors failed to establish the relative stereochemistry of this compound by ^1^H NMR data. The same compound, called preussin, was later isolated as a yellow oil ([α${]}_{D}^{25}$ + 22.0, *c* 1.0, CHCl_3_), from the mycelial cake obtained from fermentation of a fungus *Preussia* sp. [19]. By comparison of the chemical shift values of H_2_-1 and H_2_-6 of the (*S*)-and (*R*)-*O*-methylmandelate esters with those of the acetate derivative of preussin, Johnson et al. [19] determined the absolute configurations of C-2, C-3, and C-5 of preussin as 2*S*, 3*S*, and 5*R*. 

**Figure 3:**
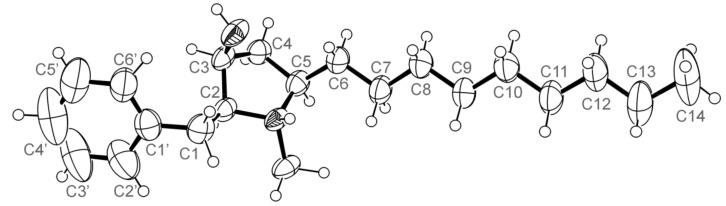
ORTEP view of a protonated **5a**.

Since we were able to obtain **5a** as a suitable crystal, X-ray analysis was carried out to determine if the absolute configurations of its stereogenic carbons were the same as those of preussin. The ORTEP view of a protonated **5a**, shown in Figure 3, not only determined the number of carbon atoms in the alkyl side chain, but also confirmed the absolute configurations of C-2, C-3, and C-5 as 2*S*, 3*S*, and 5*R*. To the best of our knowledge, this is the first X-ray crystal structure of preussin. 

Compound **5b** was isolated as a white crystal (m.p. 219–220 °C), and its molecular formula was established as C_20_H_33_NO on the basis of its (+)-HRESIMS *m*/*z* 304.2647 [M + H]^+^, (calculated 304.2640 for C_20_H_34_NO), which indicated five degrees of unsaturation. The IR spectrum showed absorption bands for hydroxyl and amine (3441 cm^−1^) and aromatic (1634, 1581, 1539 cm^−1^). The general features of the ^1^H and ^13^C NMR spectra of **5b** resembled those of **5a** (Table 2, Supplementary Materials, Figures S26, S27 and S29), except for the absence of the *N*-methyl singlet at δ_H_ 2.81 (δ_C_ 36.7), which was confirmed by its molecular formula. Moreover, H_2_-1, H-3, H-4, and H_2_-6 appeared at lower frequencies while H-2 and H-5 resonated at higher frequencies when compared to the corresponding protons in **5a**. Additionally, the chemical shift values of C-2 and C-5 in **5b** are around 10 ppm lower than those of the corresponding carbons in **5a**, while C-6 in **5b** is around 3 ppm higher than that of C-6 in **5a**. Therefore, **5b** is a *N*-demethyl analog of **5a**. This hypothesis was confirmed by COSY correlations from H-2 (δ_H_ 3.46, m; δ_C_ 65.2) to H-1a (δ_H_ 3.11, dd, *J* = 13.8, 8.0 Hz; δ_C_ 31.3), H-1b (δ_H_ 2.94, dd, *J* = 13.8, 6.6 Hz; δ_C_ 31.3) and H-3 (δ_H_ 4.09, dd, *J* = 7.9, 4.0 Hz; δ_C_ 68.7); H-3 to H-2, H-4β (δ_H_ 2.37, ddd, *J* = 13.9, 9.9, 5.5 Hz; δ_C_ 38.5) and OH-3 (δ_H_ 6.62, d, *J* = 4.0 Hz); H-5 (δ_H_ 4.09, dd, *J* = 7.9, 4.0 Hz; δ_C_ 68.7) to H-4β, H-4α (δ_H_ 1.58, dd, *J* = 13.9, 5.8 Hz; δ_C_ 38.5) and H_2_-6 (δ_H_ 1.73, m/1.64, m; δ_C_ 33.6) as well as from the methyl triplet at δ_H_ 0.86, t, (*J* = 6.8 Hz) to a brs at δ_H_ 1.26 (Table 2 and Supplementary Materials, Figures S28 and 29). This was supported by HMBC correlations from H-1a and H-1b to C-2 and C-3; H-2 to C-1; H-3 to C-2, C-5; OH-3 to C-2, C-3, C-4; H-4 to C-3, C-5 and C-6, and from H_2_-6 to C-4, C-5, C-7 (Table 2 and Supplementary Materials, Figure S30). The relative stereochemistry of the pyrrolidine ring of **5b** was established, based on NOESY experiments, to be the same as that of **5a**. The NOESY spectrum (Table 2, Supplementary Materials, Figure S31) exhibited correlations from OH-3 to H-1a, H-3 and H-4α; H-3 to H-2, OH-3 and H-4β; H-4β to H-3, H-4α and H-5, and from H-6 at δ_H_ 1.73, m to H-4α. Since **5a** can be hypothesized to derive from **5b** through *N*-methylation by SAM, it is legitimate to presume that the absolute configurations of C-2, C-3, and C-5 in **5b** should be the same as those of **5a**, i.e., 2*S*, 3*S*, 5*R*. 

Since **5b** could not be obtained as a suitable crystal for X-ray analysis, we have performed a calculated Electronic Circular Dichroism (ECD) spectrum to compare with its experimental ECD spectrum to determine unequivocally the absolute configurations of its C-2, C-3, and C-5. Experimental NOESY data of **5b**, as well as the data obtained from X-ray crystallographic and Trost’s *O*-methylmandelate method [19] of **5a**, suggest that all the pyrrolidine substituents of **5b** are directed to the same side of the ring. This formally narrows the investigation of the stereochemistry of **5b** to the enantiomer pair (2*S*, 3*S*, 5*R*) and (2*R*, 3*R*, 5*S*), combined with the two possible configurations of the secondary amine. However, comparison of ECD simulated spectra for the above-mentioned configurations of **5b**, in its neutral forms, with the experimental spectrum in methanol was inconsistent with the crystallographic configuration previously determined for its N-CH_3_ analogue (**5a**). Since this analogue, most probably a metabolic derivative of **5b** and therefore sharing the same (2*S*, 3*S*, 5*R*) configuration, precipitated and crystalized as an ammonium salt, the same was assumed for **5b** and ECD simulations were therefore redirected to its protonated (positively charged) form.

This was expected to be a major difference since the calculated SCF HOMO and LUMO orbitals show that the electronic transitions happen between the two rings. The new charge in the five-membered ring alters significantly the electron density distribution and the molecular orbitals energies. The electronic transitions associated with the hydrocarbon side-chain, however, do not involve the five-membered ring and should remain unaffected. Additionally, these transitions are predicted to be caused by wavelengths below 200 nm, hence outside the observational window.

**Figure 4:**
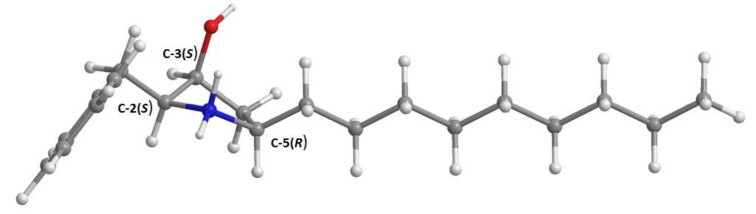
The minimal APDF (Amplitude Probability Density Function) DFT (Density Functional Theory) energy molecular model of amino-protonated **5b**, in its (2*S*, 3*S*, 5*R*) configuration. The other 17 models tested differ in the conformation of the ring and in the orientations of the hydroxyl and -CH_2_-C_6_H_6_ substituent groups.

**Figure 5:**
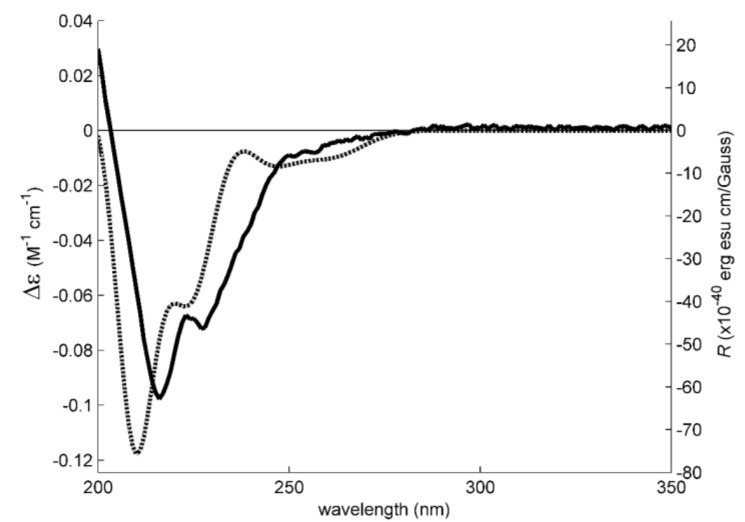
Experimental (solid line) and simulated (dotted line) methanol ECD (Electronic Circular Dichroism) spectra of **5b**. The simulated spectrum of the (2*S*, 3*S*, 5*R*) model configuration fits well the experimental data.

Models were constructed based only on the protonated (2*S*, 3*S*, 5*R*) configuration because deprotonation upon dissolution of the secondary ammonium group is expected to be negligible. The typically large pKa values (≈10) associated with these very weak acid groups in water is maintained in methanol [24]. To have a good representation of **5b**’s conformational space, the three staggered orientations of C-1 (relative to C-2) and of 3-OH (relative to C-3) were combined with two five-membered ring conformations to give 18 models. One, the lowest MM2 energy, staggered conformation of C-6 was set in all models, as well as a staggered linear conformation for the carbon chain. The orientation of the phenyl group was always determined as a result of energy minimization. To have a notion of its energetic ranking, the conformational energy of each of the 18 models was first minimized using the semi-empirical PM6 method. Since the six lowest PM6-energy conformations accounted for 98% of the conformer population, as determined by a Boltzmann distribution of computed PM6 conformational energies, these were further minimized with the Amplitude Probability Density Function (APFD) Density Functional Theory (DFT) method. The four lowest-energy APFD conformers were selected for ECD simulation, also by the Boltzmann distribution significance. Figure 4 shows the lowest energy model. The ECD transitions of the four models were calculated to a minimum wavelength of 170 nm with the TDDFT method at the same level of theory than the final minimizations (APFD, with the same basis set and solvent model). The four sets of transitions were Boltzmann weighed using the calculated populations, Gaussian broadened by 0.17 eV and added to give the averaged simulated spectrum in Figure 5. As is apparent, the fit with the experimental spectrum is good, representing well its three main negative intensities (roughly at 215, 225 and 255 nm). This evidence supports the claim that the isolated **5b** is the (2*S*, 3*S*, 5*R*) enantiomer (Figure 4) in the ammonium form.

Chrysophanic acid (**1a**), asterriquinol D dimethyl ether (**2a**), petromurin C (**2b**), kumbicin B (**2c**), kumbicin A (**2d**), 2″-oxoesterriquinol D methyl ether (**3**), kumbicin D (**4**), preussin (**5a**), preussin C (**5b**), and (3*S*, 6*S*)-3,6-dibenzylpiperazine-2,5-dione (**6**) were tested for their antibacterial activity and their minimum inhibitory concentration (MIC) and minimum bactericidal concentration (MBC) for four reference strains consisting of three multidrug-resistant isolates from the environment and one clinical isolate. In the range of concentrations tested, none of the compounds were active against Gram-negative bacteria. However, **5a** displayed an inhibitory effect against Gram-positive bacteria, with MIC values of 32 μg/mL for *S. aureus* ATCC 29213 and *E. faecalis* ATCC 29212. Moreover, **5a** consistently showed a MIC value of 32 μg/mL for both methicillin-resistant *S. aureus* (MRSA) and vancomycin-resistant enterococci (VRE) strains. Compound **5a** also displayed a MBC value of 64 μg/mL for the VRE *E. faecalis* B3/101 strain, however, it was not possible to determine the MBC for the other Gram-positive strains. This result suggests that **5a** might not only have a bacteriostatic effect but also a bactericidal action. 

**Figure 6:**
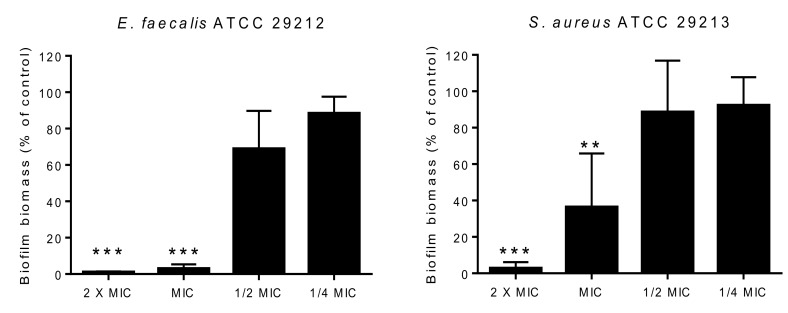
Gram-positive bacteria biofilm biomass production after 24 h of incubation with different concentrations of compound **5a**. Data are shown as Mean ± SD of the three independent experiments. One-sample *t* test: ** *p* < 0.01 and *** *p* < 0.001, significantly different from 100%. MIC, minimum inhibitory concentration.

The effect of **1a**, **2a–e**, **3**, **4**, **5a**, **b,** and **6** on biofilm formation in four reference bacterial strains was also evaluated. Four concentrations of **5a**, ranging from 2 × MIC to ¼ MIC were tested against *S. aureus* ATCC 2913 and *E. faecalis* ATCC 2912. Since it was not possible to determine a MIC of the rest of the other compounds, the previously tested highest concentration that did not inhibit bacterial growth was used. Although none of the compounds tested showed an inhibitory effect on biomass production in *P. aeruginosa* ATCC 27853, the anthraquinone chrysophanic acid (**1a**) induced significant reduction in biofilm formation (67.7 ± 8.3% of control; One-sample *t* test: * *p* < 0.05, significantly different from 100%) in *E. coli* ATCC 25922. Interestingly, **5a** also caused an almost 50% reduction of biofilm mass production (42.8 ± 32.7% of control) at a sub-inhibitory concentration in *E. coli* ATCC 25922. Although this inhibitory effect was not statistically significant, this result shows an interesting aspect in that **5a** is active against both Gram-positive and Gram-negative bacteria. In fact, among the compounds tested, only **5a** was capable of interfering with biofilm formation in *S. aureus* ATCC 29213 and *E. faecalis* ATCC 29212 at concentrations equal to or above the MIC (Figure 6), however, at lower concentrations, **5a** had no effect on the biomass production of both strains.

The tested compounds were also screened for their potential synergies with the clinically relevant antibiotics and some of them revealed small to moderate synergistic associations with antibiotic as determined by the disk diffusion method. Except **5a**, which presented an inhibition zone of 8 mm for VRE *E. faecalis* B3/101 and MRSA *S. aureus* 66/1, the rest of the tested compounds showed no inhibition zone (0 mm), when tested alone. The combination of **2a–d**, **3**, **4**, **5a**, and **6** with cefotaxime in impregnated disks resulted in a minor synergistic effect, as can be observed by a small increase in the zone of inhibition when compared with the halo of inhibition produced by cefotaxime alone in an ESBL *E. coli* strain (SA/2). In the case of VRE *E. faecalis* B3/101, all the tested compounds induced a small increase in the halo of a partial inhibition of vancomycin when compared with vancomycin alone, however, this effect was more pronounced with **5a**. On the other hand, only **2b** and **5a** revealed a synergistic effect with oxacillin against MRSA *S. aureus* 66/1. Interestingly, although **2b** alone did not exhibit the antibacterial effect, it showed a marked synergistic effect with oxacillin, increasing the halo of inhibition from 0 mm, when oxacillin was used alone, to 7 mm when tested in combination. These results were also confirmed by the checkerboard method since the combination of **5a** with vancomycin and oxacillin showed the values of ΣFIC ≤ 0.5 against VRE and MRSA isolates, respectively (Table 3). 

Interestingly, **5a** also demonstrated a strong synergistic effect with colistin, reducing MIC of colistin from 8 µg/mL to less than 0.008 µg/mL, i.e., at least 100-fold, when tested against *E. coli* 1410/1 (Table 4). Following this result, lower concentrations of **5a** were tested and it was found that a concentration of 8 µg/mL of **5a** was enough to alter *E. coli* 1410/1 resistance to colistin, thus decreasing colistin MIC to 1 µg/mL (susceptibility breakpoint of ≤0.2 µg/mL) [25].

With the exception of **2b**, the modest synergistic effects observed by the disk diffusion method were not replicated when the MIC of the antibiotics was determined in the presence of the tested compounds. The MIC of oxacillin, in a combination with **2b**, was four folds less than that of oxacillin alone (from 64 µg/mL to 16 µg/mL) when tested against MRSA *S. aureus* 66/1. Although no effect on MIC of the antibiotics was observed with any other compounds, it is likely that since the method used for determination of the MIC requires two-fold serial dilutions, the synergistic effect between the antibiotics and the test compounds might not be enough to decrease the MIC of the antibiotics by a factor of two, in case the absolute difference between the two consecutive concentrations is large. This reasoning can justify the difference in the results obtained using two different methods.

The cytotoxic effect of the ethyl acetate crude extract of *A. candidus* KUFA 0062 (200 µg/mL) and **1a**, **2a–e**, **3**, **4**, **5a**, **b**, and **6**, at only one concentration (100 µM), were tested against eight cancer cell lines, i.e., Hep G2, HT29, HCT116, A549, A375, MCF7, U251, and T98G by MTT assay. At 200 µg/mL, the crude extract significantly decreased cell viability in all cell lines tested, with higher cytotoxic effect in A375 > HT29 > HCT116 > HepG2 > A549 > T98G > MCF7 > U251 cells. At 100 µM, **5a** was found to decrease the cell viability in all cell lines tested, reaching the lowest % of cell viability in colon cancer cell lines (6.4% and 8.6% in HCT116 and HT29, respectively). Compound **5b** also decreased cell viability in all of the cell lines tested, however, it was less effective than **5a**. Except for human malignant glioma U251 and T98G cells, **2b** significantly decreased the number of viable cells in all the cell lines tested (with cell viability less than 50). Similarly, **2e** and **3** also exhibited a significant decrease of cell viability in all cell lines tested except for T98G and HepG2 for **2e** and HepG2 for **3**. Compound **2c** significantly decreased cell viability in the HepG2, HT29, HCT116, A549, and A375 cells. Compounds **1a**, **2a**, **2d**, **4** and **6**, at 100 μM, did not change cell viability in any of the cancer cell lines tested (Table 5). In view of these results, dose-response curves were constructed to determine IC_50_ values only for compounds and cell lines whose significant cytotoxic effects were observed. Doxorubicin (Dox) was used as a positive control. The IC_50_ values and respective 95% confidence intervals are summarized in Table 6. Compound **5a** was the most effective in all the cell lines tested with IC_50_ ranging from 12.3 µM in HT29 cells to 74.1 µM in U251 cells while **2b** also induced a significant decrease of cell viability with IC_50_ values ranging from 34.8 µM in H29 cells to 94.8 µM in MCF7 cells. On the other hand, **3** was more cytotoxic in HT29 cells with IC_50_ value of 43.2 µM but less cytotoxic in U251 cells with IC_50_ value of 120.2 µM. Compound **5b** showed IC_50_ values ranging from 57.2 μM (in HT29 cells) to 215.7 μM (in A549 cells). Compound **2c** and **2e** significantly decreased cell viability but with higher IC_50_ values ranging from 72.9 µM (**2c** in HCT116 cells) to 186.6 µM (**2e** in MCF7 cells). Over all, it can be observed that while **2b**, **3**, **5a,** and **5b** were more effective in HT29 cells, **2c** and **2e** were more active in HCT116 cells. This suggests that these compounds may act by different mechanisms of action since each cell line has different genetic characteristics. As to the positive control, Dox, we have observed a decrease in cell viability in all cell lines with IC_50_ values ranging from 0.05 µM (in A375 cells) to 15.4 µM (in T98G cells). Our findings are in agreement with the literature showing that HT29 was more resistant to the cytotoxic effect of Dox than the HCT116 cells [26].

The results are the mean (SD) of at least four independent experiments, each in duplicate. Significant differences (** *p* < 0.01; *** *p* < 0.001 and **** *p* < 0.0001) when compared with control cells were evaluated by one-way ANOVA, followed by the post-hoc Dunnett’s test. ^#^ Indicates significant differences when **5b** is compared with **5a**, as evaluated by a *t*-test. Compounds/extract are marked in light gray when cell viability is equal to or greater than 50% and in dark gray when cell viability is lower than 50% relative to control. n.d.—not determined.

## 3. Experimental Section

### 3.1. General Experimental Procedures

Melting points were determined on a Bock monoscope and are uncorrected. Optical rotations were measured on an ADP410 Polarimeter (Bellingham + Stanley Ltd., Tunbridge Wells, Kent, UK). Infrared spectra were recorded in a KBr microplate in a FTIR spectrometer Nicolet iS10 from Thermo Scientific (Waltham, MA, USA) with Smart OMNI-Transmission accessory (Software 188 OMNIC 8.3). ^1^H and ^13^C NMR spectra were recorded at ambient temperature on a Bruker AMC instrument (Bruker Biosciences Corporation, Billerica, MA, USA) operating at 300 or 500 and 75 or 125 MHz, respectively. High resolution mass spectra were measured with a Waters Xevo QToF mass spectrometer (Waters Corporations, Milford, MA, USA) coupled to a Waters Aquity UPLC system. A Merck (Darmstadt, Germany) silica gel GF_254_ was used for preparative TLC, and a Merck Si gel 60 (0.2–0.5 mm) was used for column chromatography.

### 3.2. Fungal Material

The strain KUFA 0062 was isolated from the marine sponge *Epipolasis* sp., which was collected, by scuba diving at a depth of 15–20 m from the coral reef at Similan Island National Park (8°39′09″ N, 97°38′27″ E), Phang-Nga province, Southern Thailand, in April 2010. The sponge was washed with 0.06% sodium hypochlorite solution for 1 min, followed by sterilized seawater three times, and then dried on sterile filter paper under a sterile aseptic condition. The sponge was cut into small pieces (5 × 5 mm), and placed on Petri dish plates containing 15 mL malt extract agar (MEA) medium with 70% seawater and 300 mg/L of streptomycin sulfate and incubated at 28 °C for 7 days. The hyphal tips emerging from the sponge pieces were individually transferred onto a MEA slant and maintained as pure culture for further identification. 

The fungus was identified as *Aspergillus candidus*, based on morphological characters as described by Varga et al. [27].This identification was supported by molecular techniques using ITS primers. DNA was extracted from young mycelia following a modified Murray and Thompson method [28]. Primer pairs ITS1 and ITS4 [29] were used for ITS gene amplification. PCR reactions were conducted on Thermal Cycler and the amplification process consisted of initial denaturation at 95 °C for 5 min, 34 cycles at 95 °C for 1 min (denaturation), at 55 °C for 1 min (annealing) and at 72 °C for 1.5 min (extension), followed by a final extension at 72 °C for 10 min. PCR products were examined by Agarose gel electrophoresis (1% agarose with 1 × TBE buffer) and visualized under UV light after staining with ethidium bromide. DNA sequencing analyses were sequenced using the dideoxyribonucleotide chain termination method [30] by Macrogen Inc. (Seoul, Korea). The DNA sequences were edited using FinchTV software and submitted into the BLAST program for alignment and compared with that of fungal species in the NCBI database (http://www.ncbi.nlm.nih.gov/). Its gene sequences were deposited in GenBank with accession numbers KX 431210. The pure cultures were deposited as KUFA 0062 at Kasetsart University Fungal Collection, Department of Plant Pathology, Faculty of Agriculture, Kasetsart University, Bangkok, Thailand.

### 3.3. Extraction and Isolation

The fungus was cultured for one week at 28 °C in five Petri dishes (i.d. 90 mm) containing 15 mL of potato dextrose agar per dish. In order to obtain the mycelial suspension, the mycelial plugs were transferred to two 500 mL Erlenmeyer flasks containing 250 mL of potato dextrose broth, and then incubated on a rotary shaker at 150 rpm at 28 °C for 7 days. Fifty 1000 mL Erlenmeyer flasks, each containing 200 g of cooked rice, were autoclaved at 121 °C for 15 min, and then inoculated with 25 mL of mycelial suspension of *A. candidus*, and incubated at 28 °C for 30 days, after which the moldy rice was macerated in ethyl acetate (25 L total) for 7 days, and then filtered with Whatman No. 1 filter paper. The ethyl acetate solutions were combined and concentrated under reduced pressure to yield 53 g of crude ethyl acetate extract, which was dissolved in 1000 mL of CHCl_3_, and filtered with Whatman No. 1 filter paper. The chloroform solution was then washed with H_2_O (3 × 500 mL) and dried with anhydrous Na_2_SO_4_, filtered and evaporated under reduced pressure to give 50 g of the crude chloroform extract, which was applied on a column of silica gel (420 g) and eluted with mixtures of petrol-CHCl_3_ and CHCl_3_-Me_2_CO, wherein the 250 mL fractions were collected as follow: Frs 1–80 (petrol-CHCl_3_, 1:1), 81–220 (petrol-CHCl_3_, 3:7), 221–350 (petrol-CHCl_3_, 1:9), 351–579 (CHCl_3_), 580–680 (CHCl_3_-Me_2_CO, 9.5:0.5), 681–730 (CHCl_3_-Me_2_CO, 9:1), 731–822 (CHCl_3_-Me_2_CO, 7:3). Frs 8–19 were combined (43.0 mg) and recrystallized in MeOH to give 17.4 mg of a yellow solid, which was identified as chrysopharic acid (**1a**) [13]. Frs 79–90 were combined (879.2 mg) and precipitated in MeOH to give 86.1 mg of clionasterol [23]. Frs. 91–118 were combined (2.49 g) and precipitated in MeOH to give 1.0 mg of palmitic acid. Frs 462–517 were combined (253.9 mg) and recrystallized in MeOH to give 31.4 mg of an orange solid of emodin (**1b**) [14]. Frs 528–583 were combined (535.8 mg) and precipitated in MeOH to give 254.4 mg of kumbicin D (**4**) [15], and the mother liquor that was crystallized in MeOH to give 11.8 mg of β-ergosterol 5,8-endoperoxide [14]. Frs 586–588 were combined (1.16 g) and recrystallized in Me_2_CO to give 345.0 mg of white crystal of asterriquinol D dimethyl ether (**2a**) [15]. Frs 589–591 were combined (780.0 mg) and precipitated in Me_2_CO to give a white solid, which was crystallized in MeOH to give 52.7 mg of kumbicin B (**2c**) [15], and the mother liquor was dried and recrystallized in Me_2_CO to give 21.6 mg of candidusin D (**2e**). Frs 610–618 were combined (870.6 mg) and crystallized in a mixture of CHCl_3_ and MeOH to give 250.6 mg of petromurin C (**2b**) [16]. The mother liquor of frs 610–618 (620.0 mg) was combined with frs. 594–609 (2.46 g) and frs 619–652 (1.29 g), and applied on a column chromatography of silica gel (130 g), and eluted with mixtures of petrol-CHCl_3_ and CHCl_3_-Me_2_CO, wherein 250 mL sfrs were collected as follow: Sfrs 1–35 (petrol-CHCl_3_, 3:7), 36–60 (petrol-CHCl_3_, 1:9), 61–100 (CHCl_3_), 101–120 (CHCl_3_-Me_2_CO, 9:1), 121–127 (CHCl_3_-Me_2_CO, 7:3). Sfrs 81–84 were combined (55.8 mg) and precipitated in Me_2_CO to give 16.3 mg of 2″-oxoasterriquinol D methyl ether (**3**) [17]. Sfrs 85–89 were combined (102.3 mg) and precipitated in CHCl_3_ to give 30.0 mg of petromurin C (**2b**). Frs 680–770 were combined (5.81 g) and applied on a column chromatography of silica gel (130 g), and eluted with mixtures of petrol-CHCl_3_, CHCl_3_-Me_2_CO, wherein 250 mL fractions were collected as follow: Sfrs 1–30 (petrol-CHCl_3_, 3:7), 31–77 (petrol-CHCl_3_, 1:9), 78–101 (CHCl_3_), 102–145 (CHCl_3_-Me_2_CO, 9:1). Sfrs 88–104 were combined (373.3 mg) and recrystallized in a mixture of CHCl_3_ and Me_2_CO to give 131.9 mg of preussin (**5a**) [18,19]. Frs 709–722 were combined (814.7 mg) and precipitated in MeOH to give 8 mg of (3*S*, 6*S*)-3,6-dibenzylpiperazine-2,5-dione (**6**). Frs 757–764 were combined (1.20 g) and precipitated in Me_2_CO to give 7 mg of kumbicin A (**2d**). Frs 789–791 were combined (1.01 g) and applied over a column chromatography of Sephadex LH-20 (20 g) and eluted with MeOH, wherein 30 sfrs of 10 mL were collected. Sfrs 24–26 were combined (48.7 mg) and precipitated in Me_2_CO to give 7.1 mg of 4-acetylamino benzoic acid (**7**) [22]. Frs 792–806 were combined (559.4 mg) and applied over a column chromatography of Sephadex LH-20 (20 g), and eluted with MeOH, wherein 40 sfrs of 10 mL were collected. Sfrs 8–14 were combined (350.4 mg) and precipitated in Me_2_CO to 25 mg of preussin C (**5b**). Frs 807–822 were combined (416.1 mg) and precipitated in a mixed CHCl_3_ and Me_2_CO to give an additional 22.1 mg of preussin C (**5b**).

#### 3.3.1. Candidusin D (**2e**)

White solid, m.p. 299–300 °C (CHCl_3_/MeOH); IR (KBr) ν_max_ 3346, 2990, 2935, 1625, 1579, 1484, 1459, 1390, 1291 cm^−1^; For ^1^H and ^13^C spectroscopic data (DMSO, 300.13 and 75.4 MHz), see Table 1; (+)-HRESIMS *m*/*z* 489.2030 (M + H)^+^ (calculated for C_28_H_29_N_2_O_6_, 489.2026).

#### 3.3.2. Preussin C (**5b**)

White solid, m.p. 219–220 °C (CHCl_3_/MeOH); [α${]}_{D}^{23}$ + 28.6 (*c* 0.04, MeOH); IR (KBr) ν_max_ 3443, 2958, 2921, 2851, 2803, 2360, 2341,1651, 1634, 1581, 1470, 1411, 1261, 1095, 1031, 803 cm^−1^; For ^1^H and ^13^C spectroscopic data, see Table 2; (+)-HRESIMS *m*/*z* 304.2647 (M + H)^+^ (calculated for C_20_H_34_NO, 304.2640).

### 3.4. Electronic Circular Dichroism

Experimental ECD spectra were obtained with a Jasco J-815 CD spectropolarimeter in a 0.1 mm cuvette. The model construction, dihedral driver search, and MM2 minimizations were done in Chem3D Ultra (Perkin-Elmer Inc., Waltham, MA, USA). All other conformational energy minimization and spectral calculations (TDDFT method) were performed with Gaussian 16W (Gaussian Inc., Wallingford, CT, USA) at the PM6 or APFD/6-311+G(2d,p) level [31] with the IEFPCM methanol solvation model. The simulated spectral lines were obtained by summation of Gaussian curves, as recommended in [32]. ECD spectra were added using Boltzmann weights derived from its minimal APFD energies [33].

### 3.5. X-ray Crystal Structure of **3** and **5a**

Single crystals were mounted on cryoloops using paratone. X-ray diffraction data were collected at room temperature with a Gemini PX Ultra equipped with CuK_α_ radiation (λ = 1.54184 Å). The structures were solved by direct methods using SHELXS-97 and refined with SHELXL-97 [34]. Non-hydrogen atoms were refined anisotropically. Hydrogen atoms were either placed at their idealized positions using appropriate HFIX instructions in SHELXL and included in subsequent refinement cycles or were directly found from difference Fourier maps and were refined freely with isotropic displacement parameters.

Full details of the data collection and refinement and tables of atomic coordinates, bond lengths and angles, and torsion angles have been deposited with the Cambridge Crystallographic Data Centre.

2″-Oxoasterriquinol D methyl ether (**3**). Crystal was triclinic, space group P-1, cell volume 1212.4(3) Å^3^ and unit cell dimensions *a* = 9.364(2) Å, *b* = 11.3095(10) Å and *c* = 12.5789(12) Å and angles α = 105.760(8)°, β = 102.321(14)° and γ = 100.259(13)° (uncertainties in parentheses). There are two molecules per unit cell with calculated density of 1.212 g/cm^−3^. Crystal has an inversion center (space group P-1) and thus the two molecules in the unit cell are enantiomers. The refinement converged to R (all data) = 15.23% and wR2 (all data) = 41.23%. (CCDC 1823071).

Preussin (**5a**). Crystal was triclinic, space group P1, cell volume 547.58(10) Å^3^ and unit cell dimensions *a* = 5.8517(5) Å, *b* = 7.0327(6) Å and *c* = 14.2627(19) Å and angles α = 79.947(10)°, β = 79.515(9)° and γ = 73.094(7)° (uncertainties in parentheses). Calculated density of 1.073 g/cm^−3^. The absolute structure was established with confidence (Flack parameter = 0.018(15)). The refinement converged to R (all data) = 3.42% and wR2 (all data) = 8.07%. (CCDC 1823438).

### 3.6. Antibacterial Activity Bioassays

#### 3.6.1. Bacterial Strains and Growth Conditions

Four reference and three multidrug-resistant bacterial strains were used in this study. The Gram-positive bacteria comprised *Staphylococcus aureus* ATCC 29213, *Enterococcus faecalis* ATCC 29212, MRSA *S. aureus* 66/1 isolated from public buses [35], and VRE *E. faecalis* B3/101 isolated from river water [36]. The Gram-negative bacteria used were *Escherichia coli* ATCC 25922, *Pseudomonas aeruginosa* ATCC 27853, a colistin-resistant *E. coli* 1418/1 strain isolated from rabbit feces, and a clinical isolate ESBL *E. coli* SA/2. Frozen stocks of all strains were grown in Mueller-Hinton agar (MH-BioKar diagnostics, Allone, France) at 37 °C. All bacterial strains were sub-cultured in MH agar and incubated overnight at 37 °C before each assay.

#### 3.6.2. Antimicrobial Susceptibility Testing

The minimum inhibitory concentration (MIC) was used for determining the antibacterial activity of each compound, in accordance with the recommendations of the Clinical and Laboratory Standards Institute (CLSI) [37]. With the exception of **6**, 10 mg/mL stock solutions of each compound were prepared in dimethylsulfoxide (DMSO—Applichem GmbH, Darmstadt, Germany). For **6**, which was less soluble in DMSO than the other compounds, a stock solution of 1 mg/mL was prepared. Two-fold serial dilutions of the compounds were prepared in Mueller-Hinton broth 2 (MHB2—Sigma-Aldrich, St. Louis, MO, USA) within the concentration range of 0.062–64 µg/mL, except for **6** for which the highest concentration tested was 32 µg/mL. The highest concentration tested was chosen in order to maintain DMSO in-test concentration below 1% as recommended by the CLSI [37]. At this concentration DMSO did not affected bacterial growth. Cefotaxime (CTX) ranging between 0.031 and 16 µg/mL was used as a control. A purity check and colony counts of the inoculum suspensions were also evaluated in order to ensure that the final inoculum density closely approximates the intended number (5 × 10^5^ CFU/mL). The MIC was determined as the lowest concentration at which no visible growth was observed. The minimum bactericidal concentration (MBC) was assessed by spreading 10 µL of culture collected from wells showing no visible growth on MH agar plates. The MBC was determined as the lowest concentration at which no colonies grew after 16–18 h incubation at 37 °C. These assays were performed in duplicate.

#### 3.6.3. Biofilm Formation Inhibition Assay

The effect of all compounds on biofilm formation was assessed using crystal violet staining as previously described [38]. Briefly, the highest concentration tested in the MIC assay was added to bacterial suspensions of 1 × 10^6^ CFU/mL prepared in Tryptic Soy broth (TSB-BioKar diagnostics, Allonne, France). When it was possible to determine a MIC, four concentrations were tested: 2 × MIC, MIC, ½ MIC, and ¼ MIC. A control without any compound as well as a negative control (TSB alone) were included. The stabilized biofilm mass was quantified after 24 h incubation at 37 °C. The absorbance was measured at 595 nm on an iMark™ microplate spectrophotometer (Bio-Rad Laboratories, Hercules, CA, USA). The background absorbance (TSB without inoculum) was subtracted from the absorbance of each sample and data are shown as a percentage of control. Three independent experiments were performed in triplicate for each experimental condition.

#### 3.6.4. Antibiotic Synergy Testing

In order to evaluate the combined effect of the compounds and clinically relevant antimicrobial drugs, a screening was conducted using the disk diffusion method, as previously described [38,39]. A set of antibiotic disks (Oxoid, Basingstoke, UK) to which the isolates were resistant was selected: cefotaxime (CTX, 30 µg) for *E. coli* SA/2, oxacillin (OX, 1 µg) for *S. aureus* 66/1, and vancomycin (VA, 30 µg) for *E. faecalis* B3/101. Antibiotic disks alone (controls) and antibiotic disks impregnated with 15 µg of each compound were placed on MH agar plates seeded with the respective bacteria. Sterile 6 mm blank paper disks (Oxoid, Basingstoke, UK) impregnated with 15 µg of each compound alone were also tested. A blank disk with DMSO was used as a negative control. MH inoculated plates were incubated for 18–20 h at 37 °C. Potential synergism was recorded when the halo of an antibiotic disk impregnated with a compound was greater than the halo of the antibiotic or compound-impregnated blank disk alone.

The potential synergy between the most promising compounds and clinically relevant antibiotics (oxacillin and vancomycin—Sigma-Aldrich, St. Louis, MO, USA) was also evaluated by the checkerboard method as previously described [38]. For this purpose multidrug-resistant strains were selected based on their resistance towards those antibiotics. The fractional concentration (FIC) was calculated as follows: FIC of drug A (FIC A) = MIC of drug A in combination/MIC of drug A alone, and FIC of drug B (FIC B) = MIC of drug B in combination/MIC of drug B alone. The FIC index was calculated as the sum of each FIC and interpreted as follows: ΣFIC ≤ 0.5, synergy; 0.5 < ΣFIC ≤ 4, no interaction; 4 < ΣFIC, antagonism [40]. When it was not possible to determine a MIC value for the test compound, the potential synergy between the compounds and clinically relevant antibiotics was evaluated by determining the antibiotic MIC in the presence of each compound. Briefly, the MIC of CTX, OX, VA, and colistin (Sigma-Aldrich, St. Louis, MO, USA) for the respective multidrug-resistant strains was determined in the presence of the highest concentration of each compound tested, unless otherwise stated, that did not affect bacterial growth when the compound was used alone. The antibiotic tested was serially diluted whereas the concentration of each compound was kept fixed. In the case of **6** the concentration used was 32 µg/mL. For all other compounds the concentration used was 64 µg/mL. Antibiotic MICs where determined as described above.

### 3.7. In Vitro Anticancer Activity Assays

#### 3.7.1. Cell Lines

Eight cancer cell lines were used to assess the anticancer activity of the extract and isolated compounds of the marine sponge-associated fungus *Aspergillus candidus* KUFA 0062. HT29, HCT116 cells were kindly provided by Prof. Carmen Jerónimo, from CI-IPO, Porto and HepG2 cells were provided by Prof. Rosário Martins, from ESTSP and CIIMAR, Porto. A375, A549, MCF7, U-251 and T98G cell lines were obtained from the European Collection of Cell Cultures. Cells were maintained as monolayer cultures in DMEM supplemented with 10% FBS, 1% antibiotic solution (100 U/mL penicillin and 100 μg/mL streptomycin), 10 mM *N*-[2-hydroxyethyl] piperazine-*N*′-[2-ethane-sulfonic acid] and 0.1 mM sodium pyruvate in a humidified incubator with 5% CO_2_ at 37 °C. Cells were trypsinized near the confluence. Stock solutions of the isolated compounds, extract and Dox were prepared in dimethyl sulphoxide (DMSO) and aliquots and kept at −20 °C. For experiments, the final concentration of DMSO in the medium was < 0.1% (*v*/*v*) and the controls received DMSO only.

#### 3.7.2. MTT Reduction Assay

To assess the effects of the isolated compounds and the extract on cell viability, cells were plated in 96-multiwell culture plates at a density of 0.1 × 10^6^ cells/mL. After cell adhesion, cells were incubated for 48 h with fresh medium containing the isolated compounds, at 100 μM, and extract, at 200 μg/mL, and cell viability was measured by MTT reduction assay as described previously [41]. Briefly, after 48 h of treatment MTT was added at a final concentration of 0.5 mg/mL and incubated for 2 h at 37 °C. The formazan crystals were dissolved in a DMSO: EtOH solution (1:1) (*v*/*v*) and the absorbance was measured at 570 nm in a microplate reader (Multiskan Ex, Labsystems, Milford, MA, USA). 

The determination of IC_50_ (half-maximal inhibitory concentration) was performed only for the isolated compounds that significantly decrease cell viability at 100 μM. For this, cancer cell lines were incubated with different concentrations of isolated compounds (0–200 μM) or Dox (0.001–10 μM), as a positive control, and the MTT assay was performed as described above. The IC_50_ values were determined using GraphPad Prism v6.0 software (GraphPad Software, La Jolla, CA, USA). This software was also used to conduct the statistical analysis, using a one-way ANOVA, followed by the post-hoc Dunnett’s test. The normality and homogeneity of variance were confirmed by the Shapiro-Wilk test and Levine test, respectively. The significance level was set at the conventional 95%.

## 4. Conclusions

Most of the investigations of the secondary metabolites of the fungus *Aspergillus candidus* were carried out with its terrestrial strains. Except for a chlorine containing flavone antifungal antibiotic chlorflavonin, the secondary metabolites produced by this fungal species were *p*-terphenyl and *bis*-indolyl benzenoids derivatives, some of which immunosuppressive activity, cytotoxicity against sea urchin embryos and various tumor cell lines. Interestingly, to the best of our knowledge, only two marine-derived *A. candidus* were investigated chemically. While *A. candidus*, isolated from the marine sediment furnished *p*-terphenyl derivatives, the strain isolated from the gut of sea urchin produced a different group of metabolites, i.e., spiculisporic derivatives. This fact has prompted us to investigate the secondary metabolites from a marine-sponge associated strain of *A. candidus* to verify if the marine host can influence the type of secondary metabolites produced by this fungus. Our results were very interesting since we isolated, besides the commonly found fungal metabolites such as palmitic acid, ergosterol-5,8-endoperoxide, the anthraquinones chrysophanic acid and emodin, a diketopiperazine derivative, and *bis*-indolyl derivatives that are the chemical signature of this fungus, one previously reported hydroxypyrrolidine alkaloid preussin (**5a**) and a new congener, preussin C (**5b**). Although preussin (**5a**) was isolated from *A. ochraceus* and other fungi (*Preussia* sp. and *Simplicillium lanosoniveum*), this is the first report of the isolation of hydroxypyrrolidine alkaloids from *A. candidus*. From the biological perspective, it is interesting to note that although most of the tested secondary metabolites exhibited a cytotoxic effect against eight human cancer cell lines, their activity varied depending on the cell lines. The most notable aspect was that the presence of the *N*-methyl group on the pyrrolidine ring in preussin (**5a**) was always crucial in both antibacterial and cytotoxic activities. This was demonstrated by the effectiveness of preussin (**5a**) to decrease cell viability when compared to the *N*-demethyl analogue, preussin C (**5b**). For the antibacterial activity assay, it is important to point out that, among the compounds tested, only preussin (**5a**) stood out since it displayed a relevant inhibitory effect against Gram-positive bacteria as well as methicillin-resistant *S. aureus* (MRSA) and vancomycin-resistant enterococci (VRE) strains. Moreover, preussin (**5a**) was not only able to interfere with biofilm formation in *S. aureus* ATCC 29213 and *E. faecalis* ATCC 29212 at concentrations equal to or above the MIC but also showed synergistic effect with vancomycin and oxacillin against VRE and MRSA isolates and a strong synergistic effect with colistin when tested against *E. coli* 1410/1. Therefore, our study reveals that preussin (**5a**) can represent a new model for the development of a new class of antibiotic and anticancer agents.

## Figures and Tables

**Table 1:** ^1^H and ^13^C NMR (DMSO, 300.13 and 75.4 MHz) and HMBC assignment for **2e**.

Position	δ_C_, Type	δ_H_, (*J* in Hz)
1, 2, 4, 5	147.6, C	-
3, 6	120.0, C	-
NH-1′, 1″	-	11.15, d (2.2)
2′, 2″	126.0, CH	7.43, d (2.5)
3′, 3″	106.7, C	-
4′, 4″	102.1, CH	6.88, d (2.5)
5′, 5″	153.1, C	-
6′, 6″	110.9, CH	6.78, dd (8.8, 2.5)
7′, 7″	111.9, CH	7.33, d (8.8)
8′, 8″	131.0, C	-
9′, 9″	127.7	-
OCH_3_-1, 2, 4, 5	60.3, CH_3_	3.46, s
OCH_3_-5, 5′	55.3, CH2	3.72, s

**Table 2:** ^1^H and ^13^C NMR of **5a** (DMSO, 300.13 and 75.4 MHz) and **5b** (DMSO, 300.13 and 75.4 MHz) and HMBC and NOESY assignments for **5b**.

5a (DMSO, 300.13 and 75.4 MHz)			5b (DMSO, 500.13 and 125.4 MHz)				
Position	δ_C_, Type	δ_H_, (*J* in Hz)	δ_C_, Type	δ_H_, (*J* in Hz)	COSY	HMBC	NOESY
1a	30.6, CH_2_	3.69, dd (13.0, 9.4)	31.3, CH_2_	3.11, dd (13.8, 8.0)	H-1b, 2	C-2, 3	H-1b, 2′, 6′
b		3.13, dd (12.7, 4.8)		2.94, dd (13.8, 6.6)	H-1a, 2	C-2, 3	H-1a
2	76.0, CH	3.09, q (4.7)	65.5, CH	3.46, m	H-1b, 1b, 3	C-1	H-3
3	68.6, CH	4.22, m	68.7, CH	4.09, dd (7.9, 4.0)	H-2, 4β, OH-3	C-2, 5	H-2, H-4β, OH-3
4α	38.8, CH_2_	2.06, ddd (14.8, 9.3, 2.1)	38.5, CH_2_	1.58, dd (13.9, 5.8)	H-3, 5	C-3, 5, 6	H-3, 4β, 5
β		2.69, ddd (15.0, 8.2, 8.2)		2.37, ddd (13.9, 9.9, 5.5)	H-5	C-2, 3, 5, 6	H-4α, H-3
5	69.3, CH	2.92,m	57.6, CH	3.41, m	H-4β, 4α, 6	C-4, 6, 7	
6	30.1, CH_2_	2.25, m	33.6, CH_2_	1.73, m	H-5	C-4, 5, 7	H-4α
		1.95, m		1.64, m	H-5	C-4, 5, 7	
7	26.5, CH_2_	1.40, m	26.1, CH_2_	1.26, brs			
8	26.5, CH_2_	1.22, brs	28.6, CH_2_	1.26, brs			
9	29.4, CH_2_	1.22, brs	28.9, CH_2_	1.26, brs			
10	29.2, CH_2_	1.22, brs	28.8, CH_2_	1.26, brs			
11	29.2, CH_2_	1.22, brs	28.7, CH_2_	1.26, brs			
12	29.4, CH_2_	1.22, brs	31.6, CH_2_	1.26, brs			
13	22.7, CH_2_	1.22, brs	22.1, CH_2_	1.26, brs			
14	14.1, CH_3_	0.88, t (6.5)	14.0, CH_3_	0.86, t (6.8)	H-13	C-12, 13	
1′	136.1, C	-	137.4, C	-			
2′	129.4, CH	7.31, m	129.1, CH	7.31, m		C-1	H-1a, 1b, H2
3′	128.9, CH	7.35, m	128.5, CH	7.34, m		C-1	
4′	127.2, CH	7.25, m	126.6, CH	7.26, m		C-2′, 6′	
5′	128.9, CH	7.31, m	128.5, CH	7.34, m		C-1	
6′	129.4, CH	7.35, m	129.1, CH	7.31, m		C-1	H-1a, 1b, H2
N-CH_3_	36.7, CH_3_	2.81, s	-	-			
OH-3	-	5.12, d (11.7)	-	5.62, d (4.0)	H-3	C-2, 3, 4	H-1a, 3, 4β

**Table 3:** Fractional inhibitory concentration (FIC) index of **5a** in combination with clinically relevant antibiotics obtained by the checkerboard method.

Bacterial Strain	5a-Van		5a-Ox	
	ΣFIC	Activity	ΣFIC	Activity
*E. faecalis* B3/101 (VRE)	0.4	S	-	-
*S. aureus* 66/1 (MRSA)	-	-	0.2	S

S = synergism; VAN = vancomycin; OX = oxacillin.

**Table 4:** Combined effect of colistin with **5a** against colistin resistant *E. coli* strain 1410/1. MICs for colistin are expressed in µg/mL.

Compound	µg/mL of 5a + Colistin							
**5a**	**0**	**1**	**2**	**4**	**8**	**16**	**32**	**64**
Colistin (MIC)	8 ^R^	8 ^R^	8 ^R^	4 ^R^	1 ^S^	0.016 ^S^	<0.008 ^S^	<0.008 ^S^

MIC = minimum inhibitory concentration; ^R^ = resistant; ^S^ = sensitive.

**Table 5:**
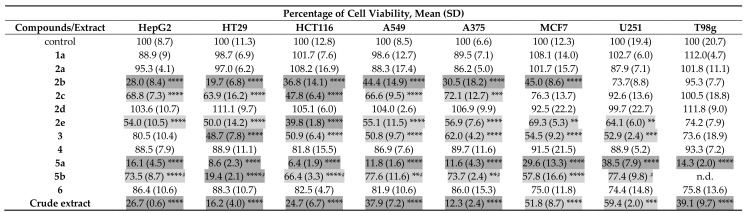
Percentage of cell viability relative to control of **1a**, **2a–e**, **3**, **4**, **5a**, **b**, **6** (100 µM) and crude extract of *A. candidus* KUFA 0062 (200 µg/mL) in eight human cancer cell lines after 48 h of incubation as assessed by MTT assay.

The results are the mean (SD) of at least four independent experiments, each in duplicate. Significant differences (** *p* < 0.01; *** *p* < 0.001 and **** *p* < 0.0001) when compared with control cells were evaluated by one-way ANOVA, followed by the post-hoc Dunnett’s test. ^#^ Indicates significant differences when **5b** is compared with **5a**, as evaluated by a *t*-test. Compounds/extract are marked in light gray when cell viability is equal to or greater than 50% and in dark gray when cell viability is lower than 50% relative to control. n.d.—not determined.

**Table 6:** IC_50_ values (half-maximal inhibitory concentration) and respective 95% confidence intervals of compounds tested in eight human cancer cell lines, determined by the MTT assay.

IC_50_ (95% CI)								
Compounds (µM)	HepG2	HT29	HCT116	A549	A375	MCF7	U251	T98G
**2b**	56.3	34.8	60.8	84.1	82.8	94.8	n.d.	n.d.
	(37.9–83.7)	(22.0–55.0)	(41.6–88.8)	(60.3–117.1)	(50.5–123.8)	(63.2–142.3)		
**2c**	123.8	113.7	72.9	109.0	146.4	n.d.	n.d.	n.d.
	(86.4–177.5)	(78.9–163.8)	(56.0–94.9)	(66.2–179.5)	(102.3–209.4)			
**2e**	118.9	111.6	73.2	105.8	123.1	186.8	212.5	n.d.
	(88.9–159.0)	(85.9–145.0)	(59.3–90.4)	(72.8–153.7)	(91.9–164.9)	(155.1–224.8)	(156.2–289.2)	
**3**	n.d.	43.2	64.1	85.2	109.8	99.7	120.2	n.d.
		(28.3–65.7)	(42.9–95.7)	(61.9–117.3)	(76.4–157.6)	(70.8–143.0)	(81.2–177.7)	
**5a**	43.2	12.3	29.8	47.9	38.5	53.6	74.1	50.4
	(30.7–60.8)	(10.1–15.5)	(19.1–46.6)	(33.2–69.0)	(29.8–49.7)	(39.4–72.9)	(54–0–101.6)	(32.0–79.4)
**5b**	153.4	57.2	124.8	215.7	148.5	128.8	128.6	–
	(96.2–244.7)	(41.7–78.4)	(94.9–164.1)	(161.4–288.1)	(103.7–212.4)	(94.5–175.5)	(86.7–190.8)	
**Dox**	0.12	0.63	0.29	0.24	0.05	0.36	1.11	15.4
	(0.07–0.22)	(0.26–1.12)	(0.16–0.54)	(0.13–0.08)	(0.03–0.08)	(0.16–0.84)	(0.43–2.85)	(10.4–22.9)

n.d.: IC_50_ not determined because no cytotoxicity was observed at 100 µM. IC_50_ values (in µM) are the mean of at least four independent experiments, each in duplicate. Doxorubicin (Dox) was used.

## Supplementary Materials

The following are available online at http://www.mdpi.com/1660-3397/16/4/119/s1, Figure S1: Structures of palmitic acid, clionasterol and ergosterol-5,8-endoperoxide, isolated from the marine sponge-associated fungus *Aspergillus candidus* KUFA0062, Figures S2–S35: 1D and 2D NMR spectra of isolated compounds, Table S1: ^1^H NMR data of **2a–d** (DMSO, 300.13 MHz), Table S2:^ 13^C NMR data of **2a–d** (DMSO, 75.3 MHz), Table S3: Comparison of ^1^H and ^13^C NMR data of **3** (DMSO, 300.13 and 75.4 MHz) and 2″-oxoasterriquinol D methyl ether (CDCl_3_, 300 MHz), Table S4: ^1^H and ^13^C NMR data of **4** (kumbicin D) (300.13 MHz and 75.4 MHz).
