# Supplementary material for: Bis-Indolyl Benzenoids, Hydroxypyrrolidine Derivatives and Other Constituents from Cultures of the Marine Sponge-Associated Fungus Aspergillus candidus KUFA0062
Source: Mar Drugs. 2018 Apr 6;16(4):119. doi: 10.3390/md16040119 (PMC5923406; doi:10.3390/md16040119)
Supplement: Supplementary file 1 [file marinedrugs-16-00119-s001.pdf]

Supplementary Materials

Bis-Indolyl Benzenoids, Hydroxypyrrolidine Derivatives and Other Constituents from Cultures of the Marine Sponge-Associated Fungus *Aspergillus candidus* KUFA0062

Suradet Buttachon[1, 2], Alice A. Ramos[1, 2], Ângela Inácio[1, 2], Tida Dethoup[3], Luís Gales[1, 4], Michael Lee[5], Paulo M. Costa[1, 2], Artur M. S. Silva[6], Nazim Sekeroglu[7], Eduardo Rocha[1, 2], Madalena M. M. Pinto[2, 8], José A. Pereira[1, 2*], Anake Kijjoa[1, 2*]

[1] *ICBAS*-Instituto de Ciências Biomédicas Abel Salazar, Rua de Jorge Viterbo Ferreira, 228, 4050-313 Porto, Portugal. E-mail: ankijjoa@icbas.up.pt (A.K.), erocha@icbas.up.pt (E.R.), jpereira@icbas.up.pt (J.A.P), pmcosta@icbas.up.pt (P.M.C).

[2] Interdisciplinary Centre of Marine and Environmental Research (CIIMAR), Terminal de Cruzeiros do Porto de Lexões, Av. General Norton de Matos s/n, 4450-208, Matosinhos, Portugal. E-mail: nokrari_209@hotmail.com (S.B), ramosalic@gmail.com (A.A.R), angelainacio@gmail.com (A.I).

[3] Department of Plant Pathology, Faculty of Agriculture, Kasetsart University, Bangkok 10240, Thailand. E-mail: tdethoup@yahoo.com.

[4] Instituto de Biologia Molecular e Celular (i3S-IBMC), Universidade do Porto, Rua de Jorge Viterbo Ferreira, 228, 4050-313 Porto, Portugal. E-mail: lgales@ibmc.up.pt.

[5] Department of Chemistry, University of Leicester, University Road, Leicester LE 7 RH, UK. E-mail: ml34@leicester.ac.uk.

[6] Departamento de Química & QOPNA, Universidade de Aveiro, 3810-193 Aveiro, Portugal. E-mail: artur.silva@ua.pt.

[7] Medicinal and Aromatic Plant Programme, Plant and Animal Sciences Department, Vocational School, Kilis 7 Aralık University, 79000, Kilis, Turkey. E-mail: nsekeroglu@gmail.com.

[8] Laboratório de Química Orgânica, Departamento de Ciências Químicas, Faculdade de Farmácia, Universidade do Porto, Rua de Jorge Viterbo Ferreira, 228, 4050-3 13 Porto, Portugal. E-mail: madalena@ff.up.pt

Figure S1. Structures of palmitic acid, clionasterol and ergosterol 5,8-endoperoxide isolated from *Aspergillus candidus* KUFA 006231.

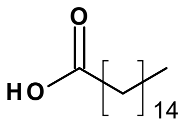

Palmitic acid

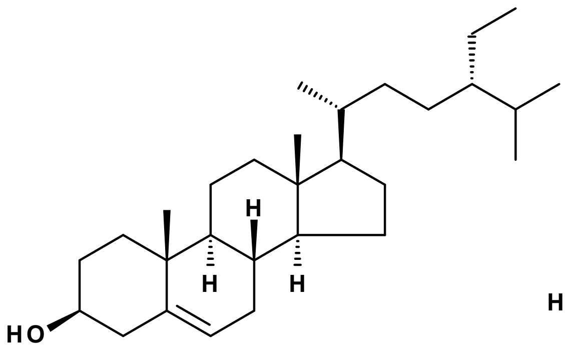

Clionasterol

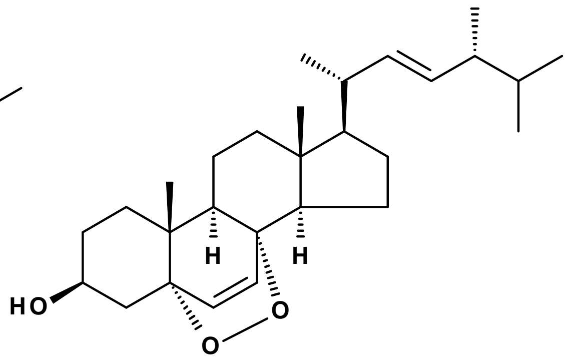

Ergosterol-5,8-endoperoxide

Figure S2. ^{1}H NMR spectrum of clionasterol ($CDCl_3$, 300.13 MHz).

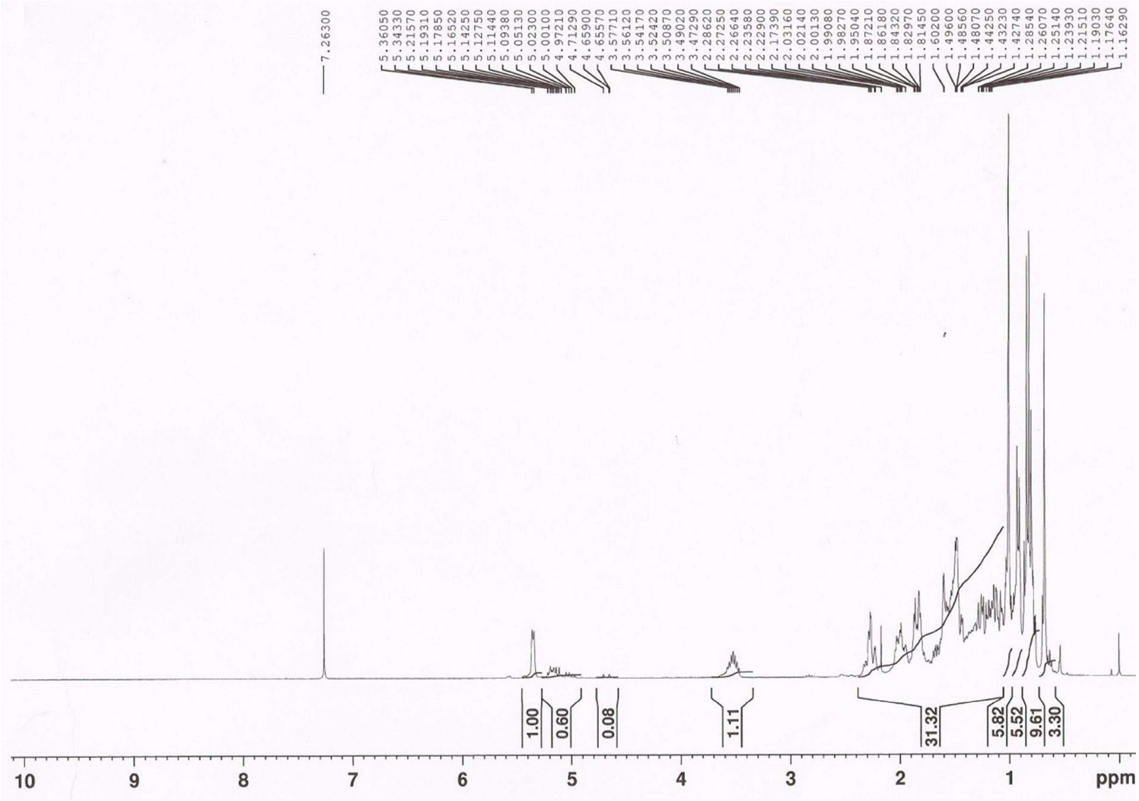

Figure S3. ^{13}C NMR spectrum of clionasterol ($CDCl_3$, 75.4 MHz).

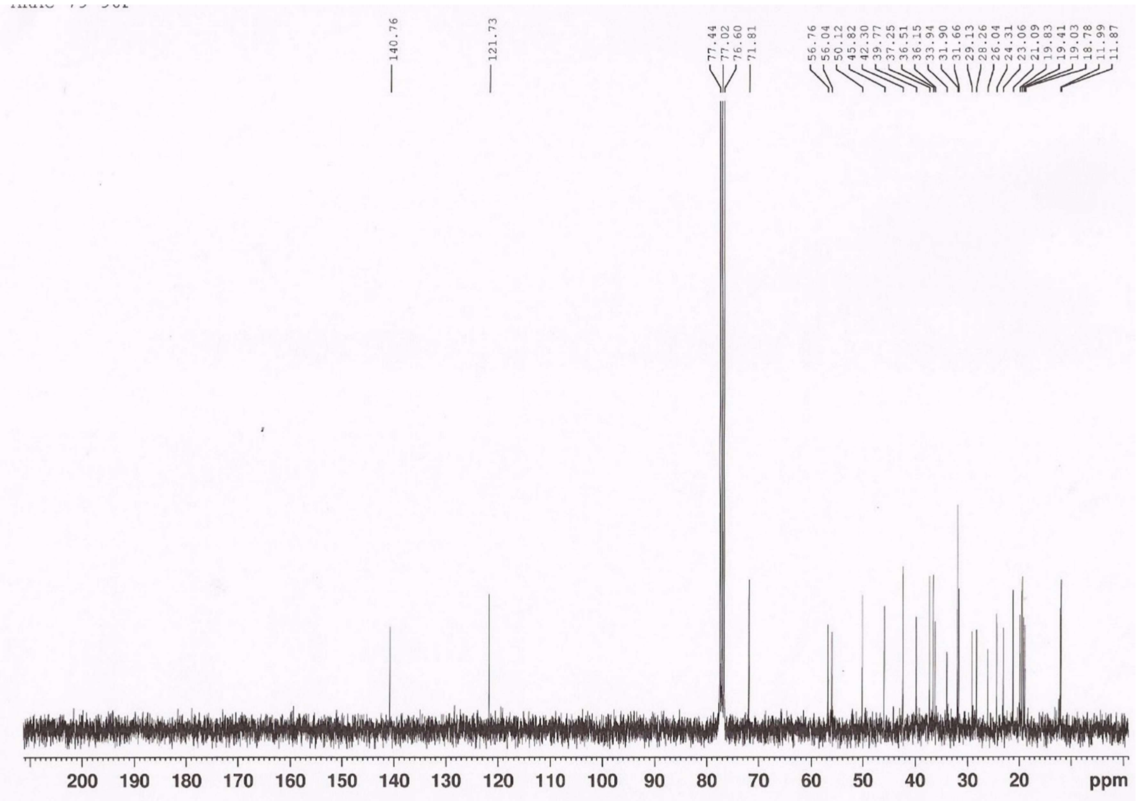

Figure S4. ^{1}H NMR spectrum of ergosterol-5,8-endoperoxide ($CDCl_3$, 300.13 MHz).

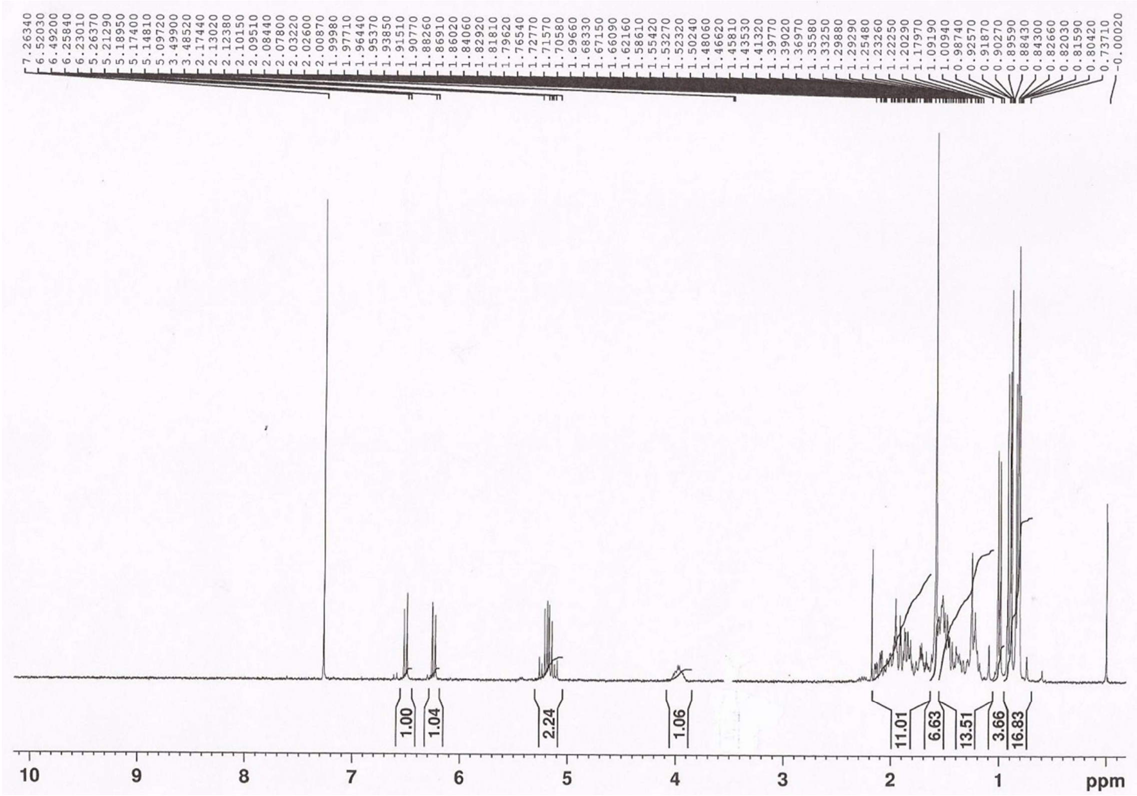

Figure S5. ^{13}C NMR spectrum of ergosterol-5,8-endoperoxode ($CDCl_3$, 75.4 MHz).

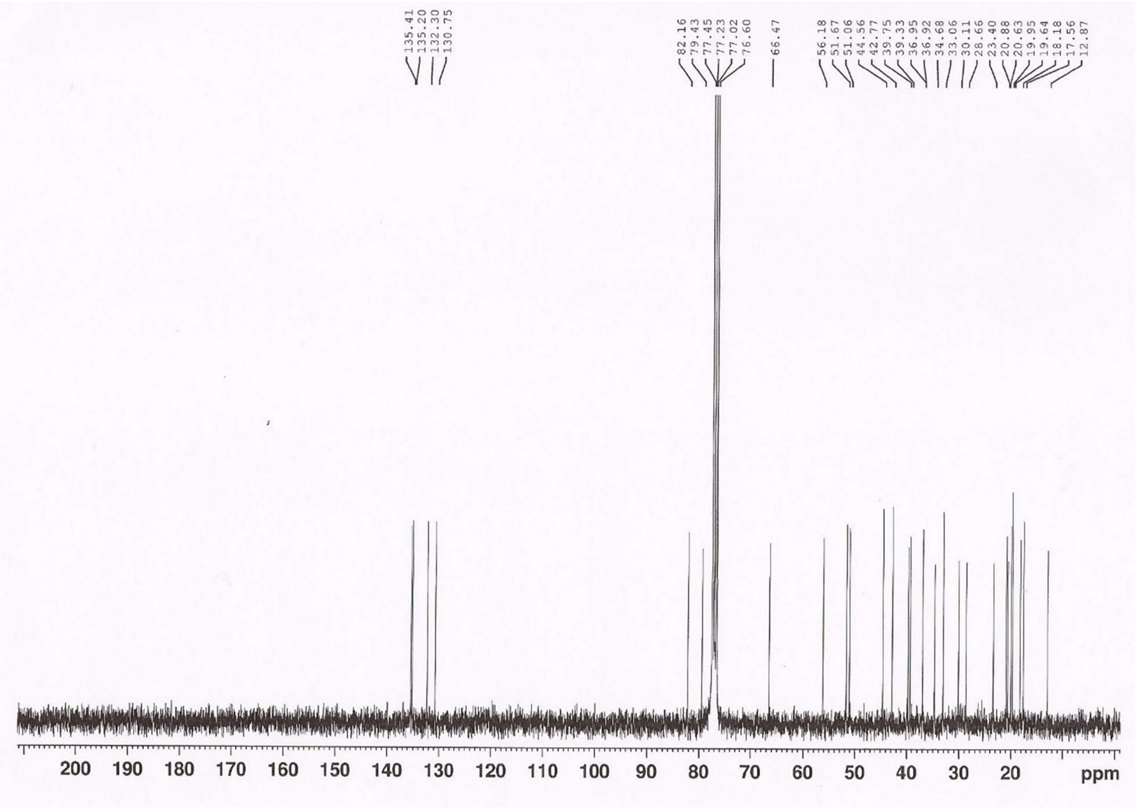

Figure S6. ^{1}H NMR spectrum of chrysophanic acid (**1a**) ($CDCl_3$, 300.13 MHz).

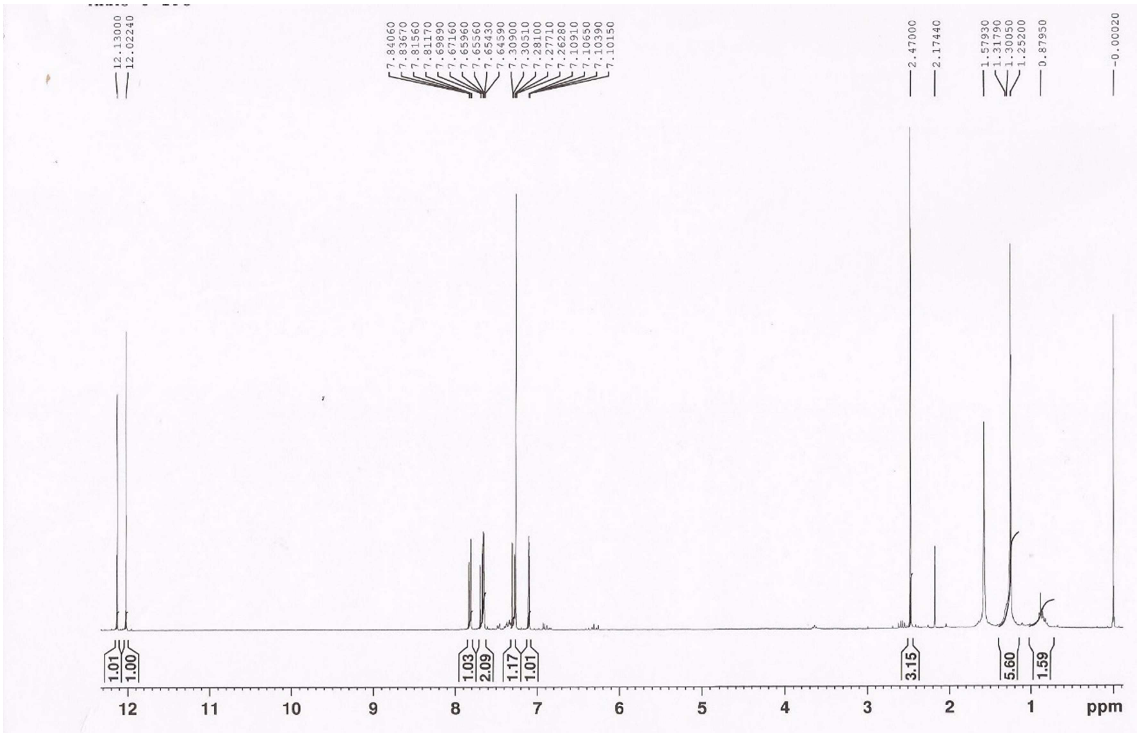

Figure S7. ^{13}C NMR spectrum of chrysophanic acid (**1a**) ($CDCl_3$, 75.4 MHz).

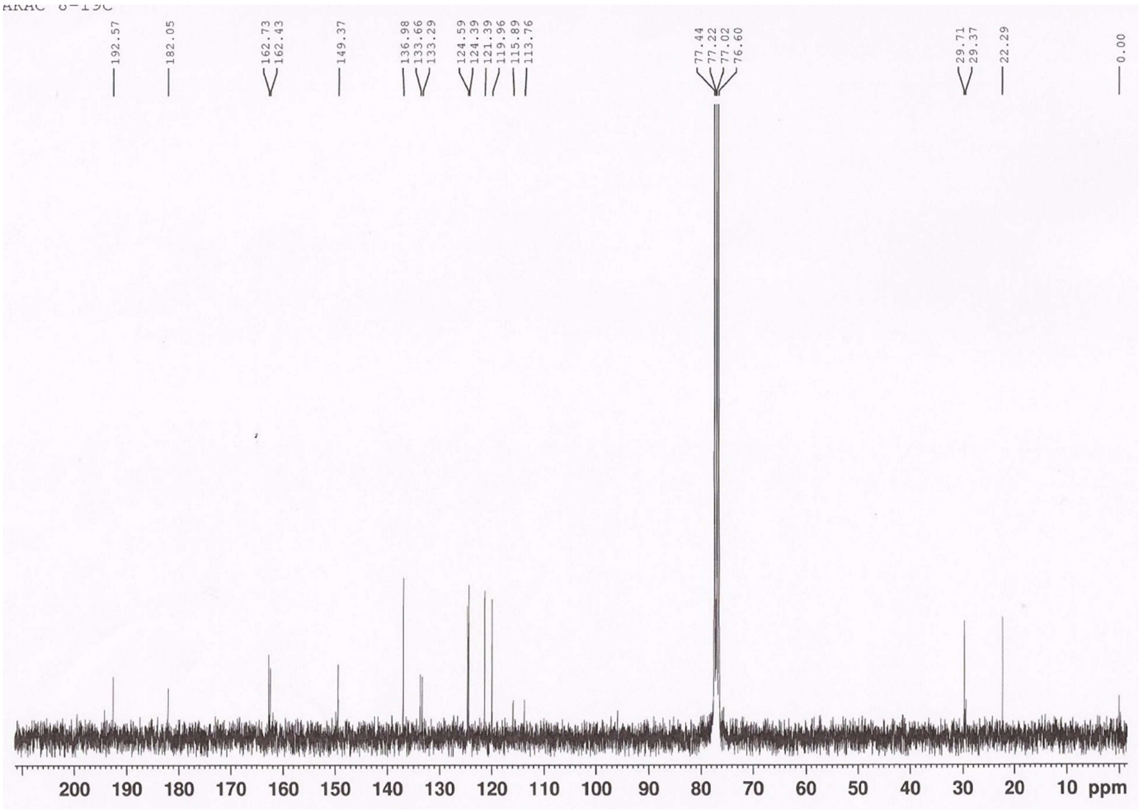

Figure S8. ^{1}H NMR spectrum of emodin (**1b**) (DMSO, 300.13 MHz).

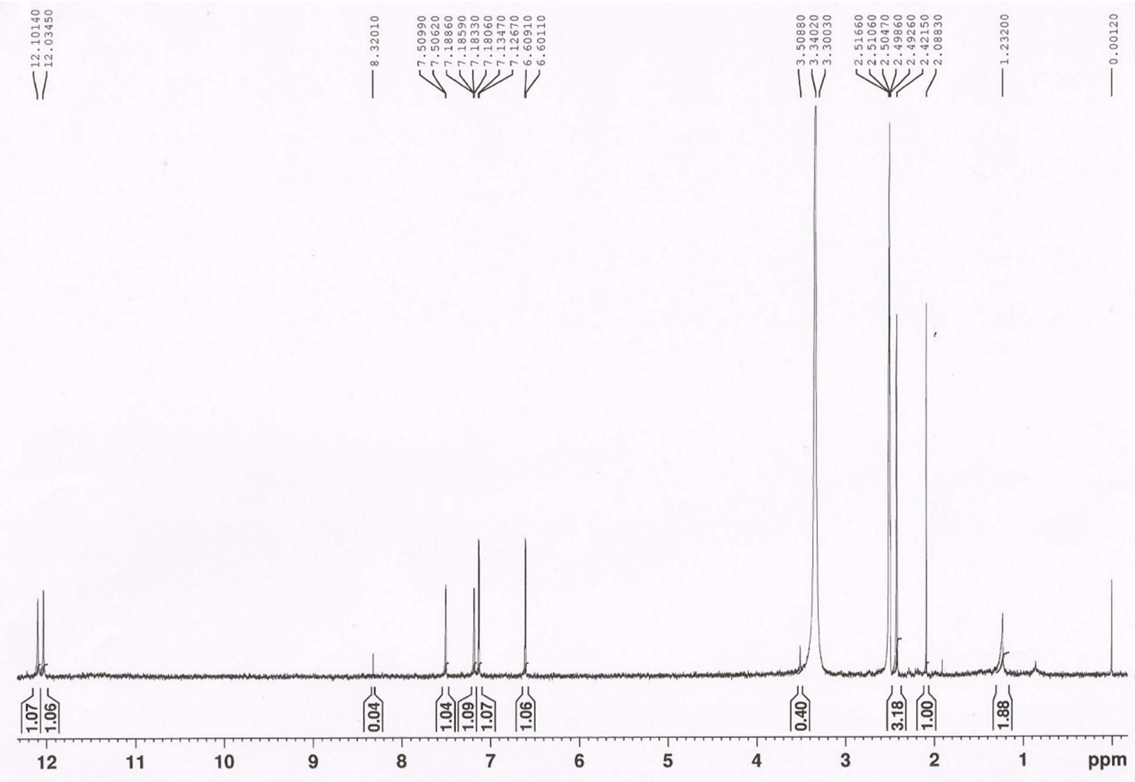

Figure S9. ^{13}C NMR spectrum of emodin (**1b**) (DMSO, 75.4 MHz).

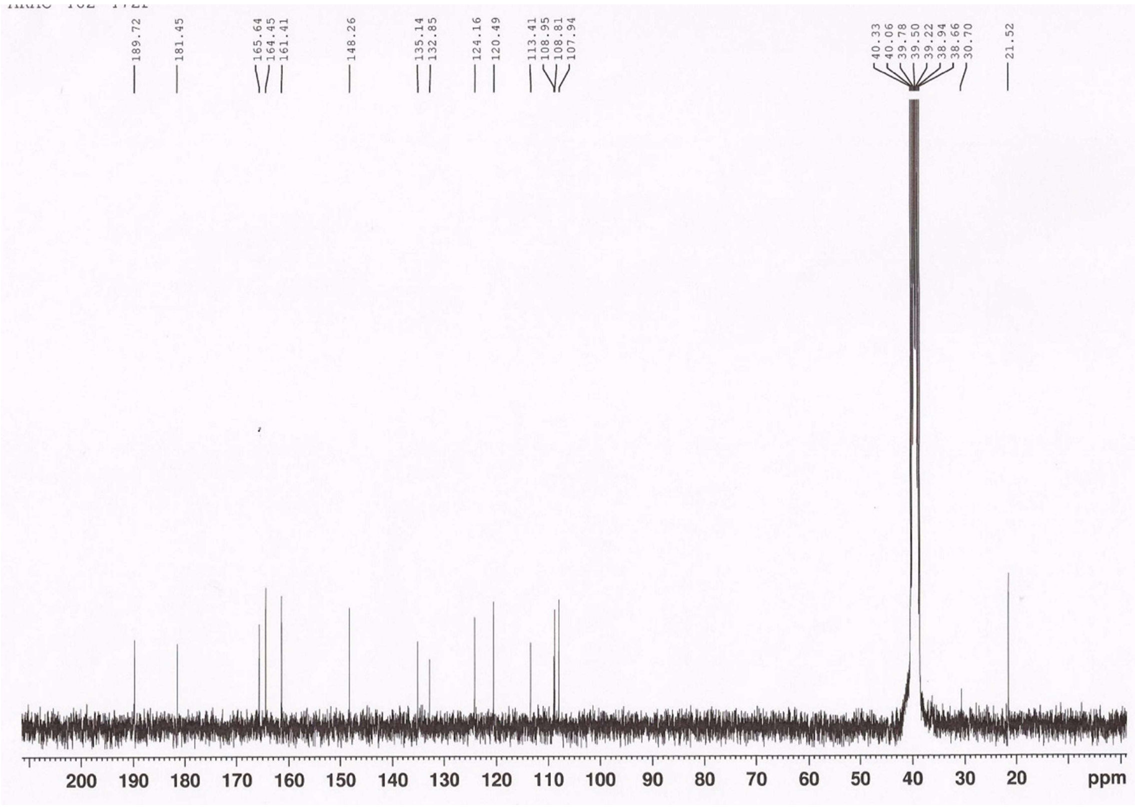

Figure S10. ^{1}H NMR spectrum of asterriquinol D dimethylether (**2a**) (DMSO, 300.13 MHz).

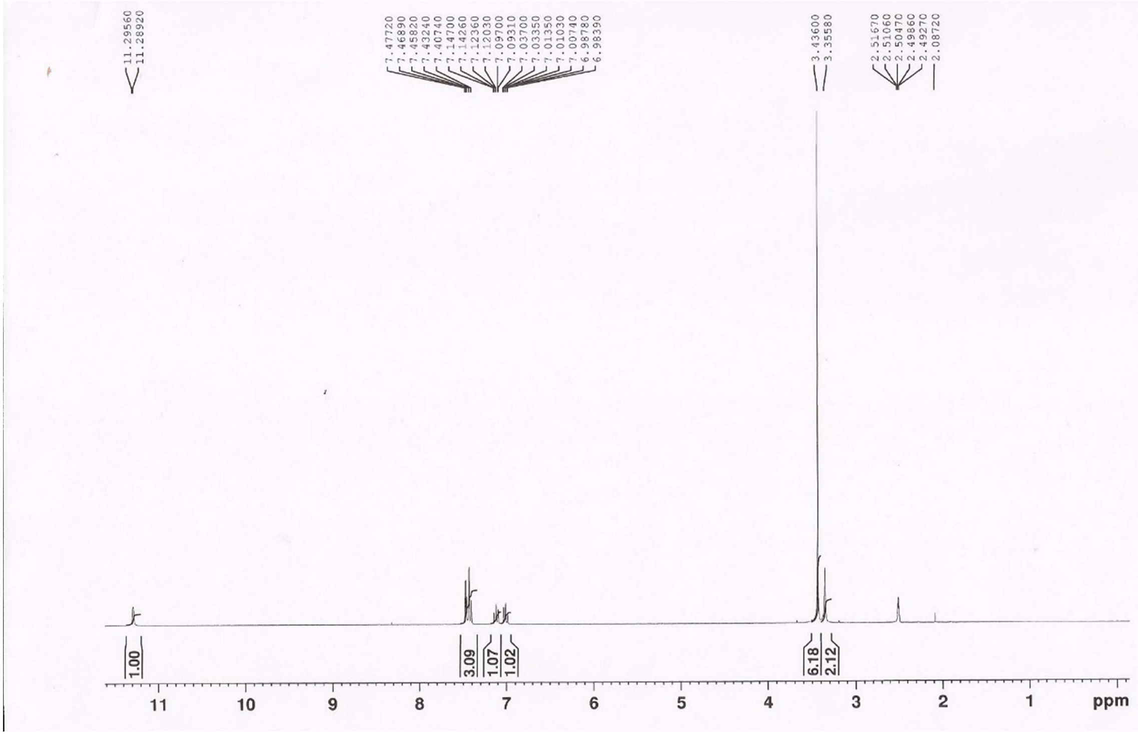

Figure S11. ^{13}C NMR spectrum asterriquinol D dimethylether (**2a**) (DMSO, 75.4 MHz).

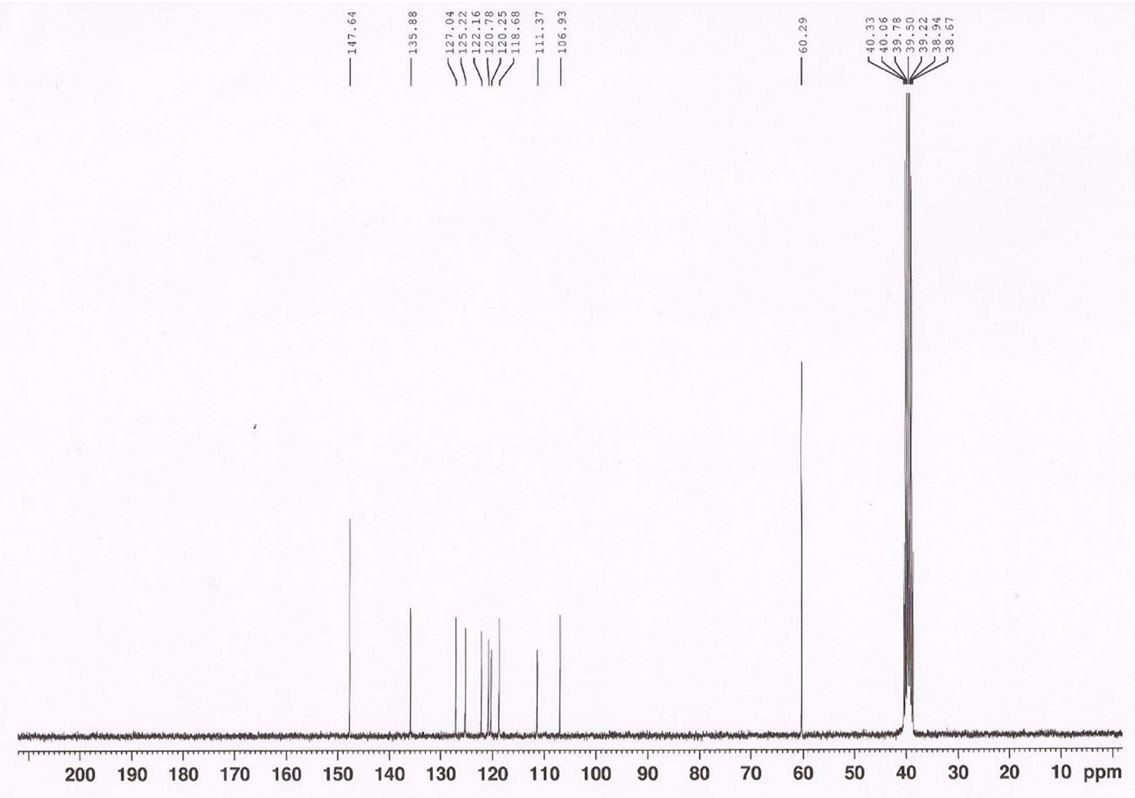

Figure S12. ^{1}H NMR spectrum of petromurin C (**2b**) (DMSO, 300.13 MHz).

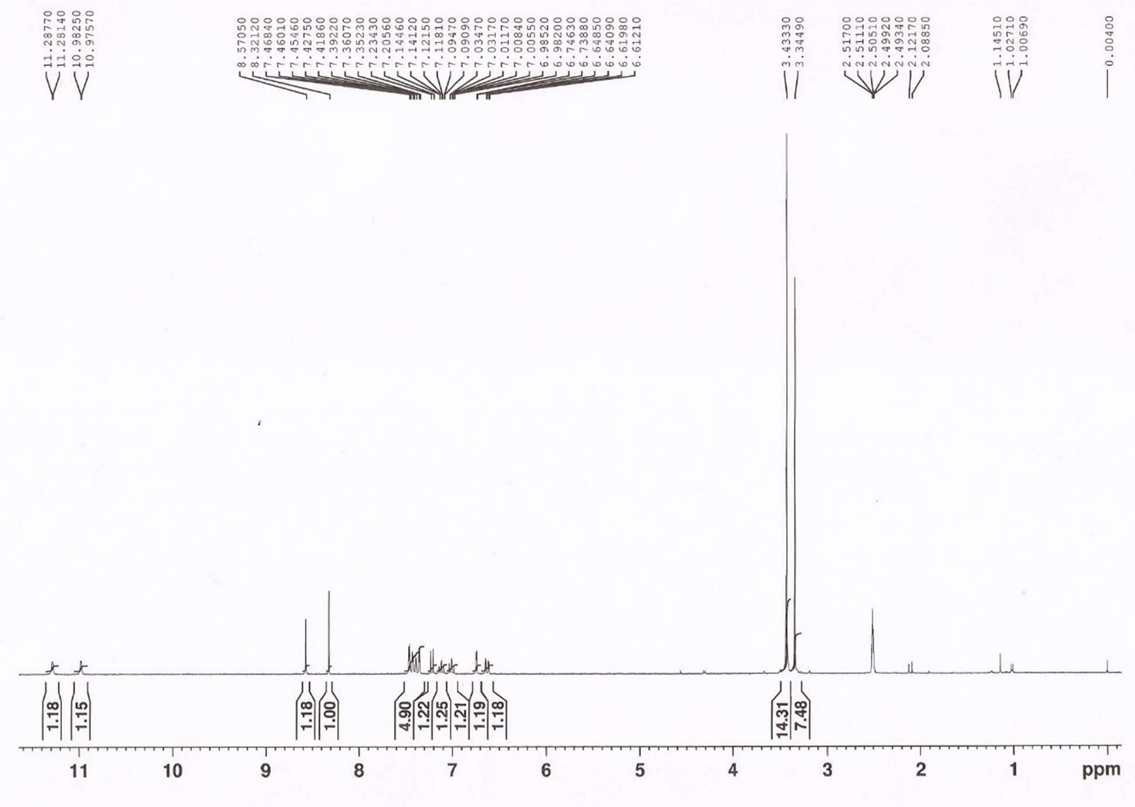

Figure S13. ^{13}C NMR spectrum petromurin C (**2b**) (DMSO, 75.4 MHz).

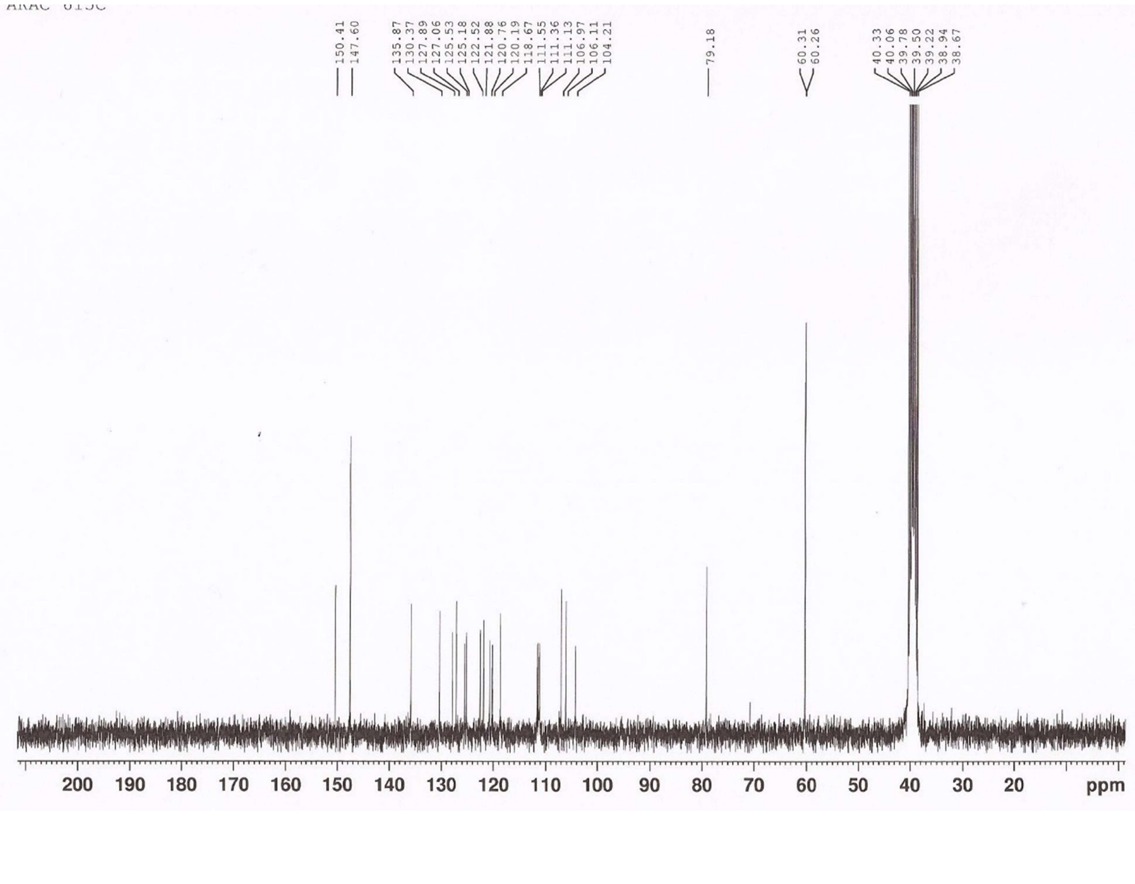

Figure S14. ^{1}H NMR spectrum of kumbicin B (**2c**) (DMSO, 300.13 MHz).

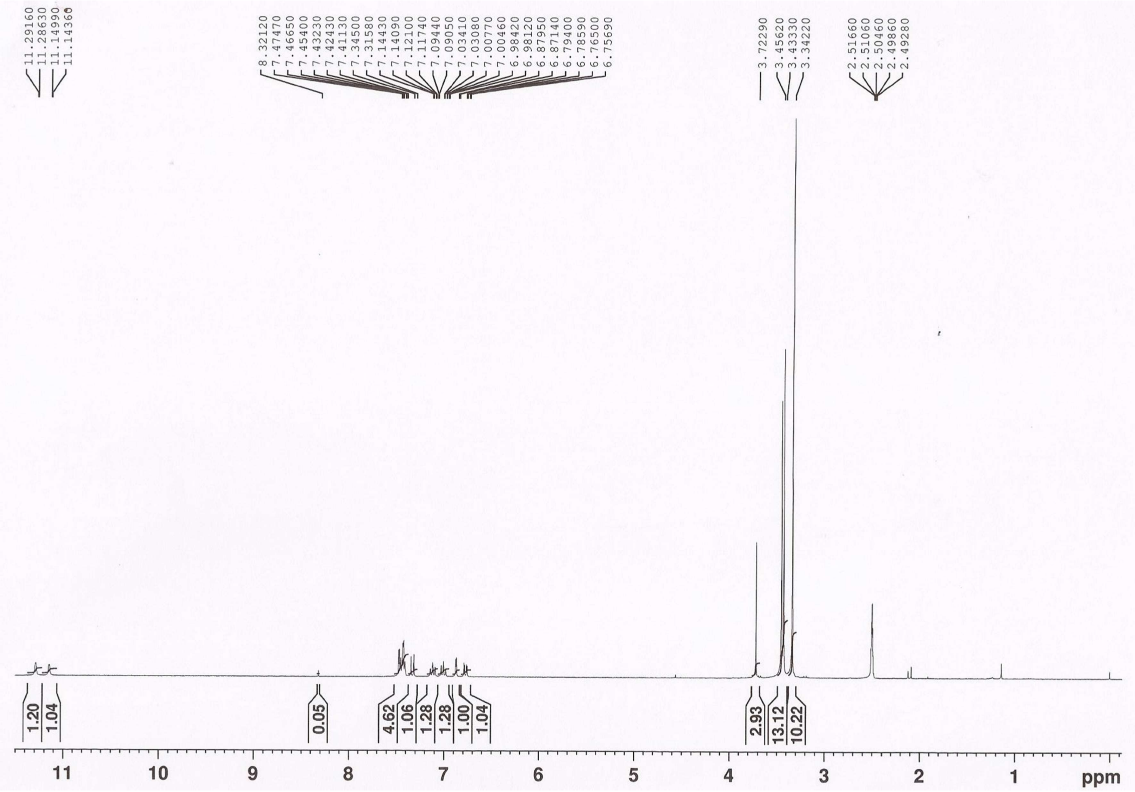

Figure S15. ^{13}C NMR spectrum kumbicin B (**2c**) (DMSO, 75.4 MHz).

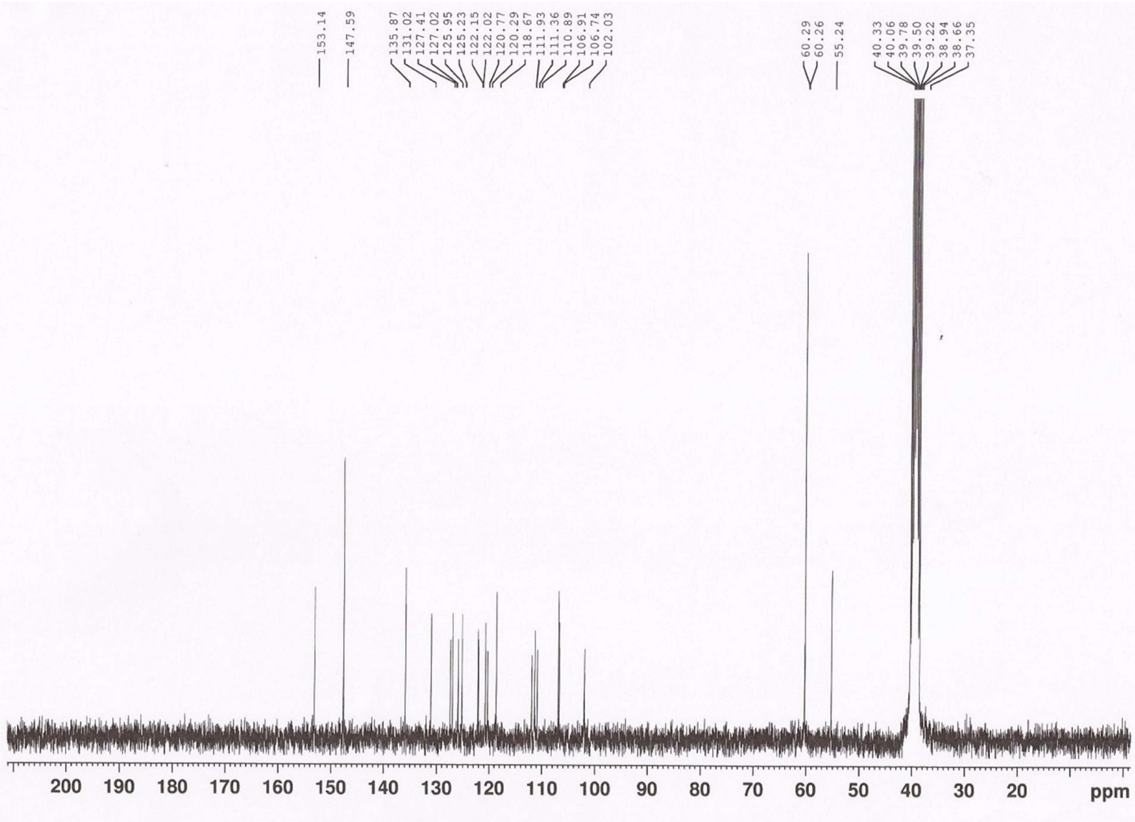

Figure S16. ^{1}H NMR spectrum of kumbicin A (**2d**) (DMSO, 300.13 MHz).

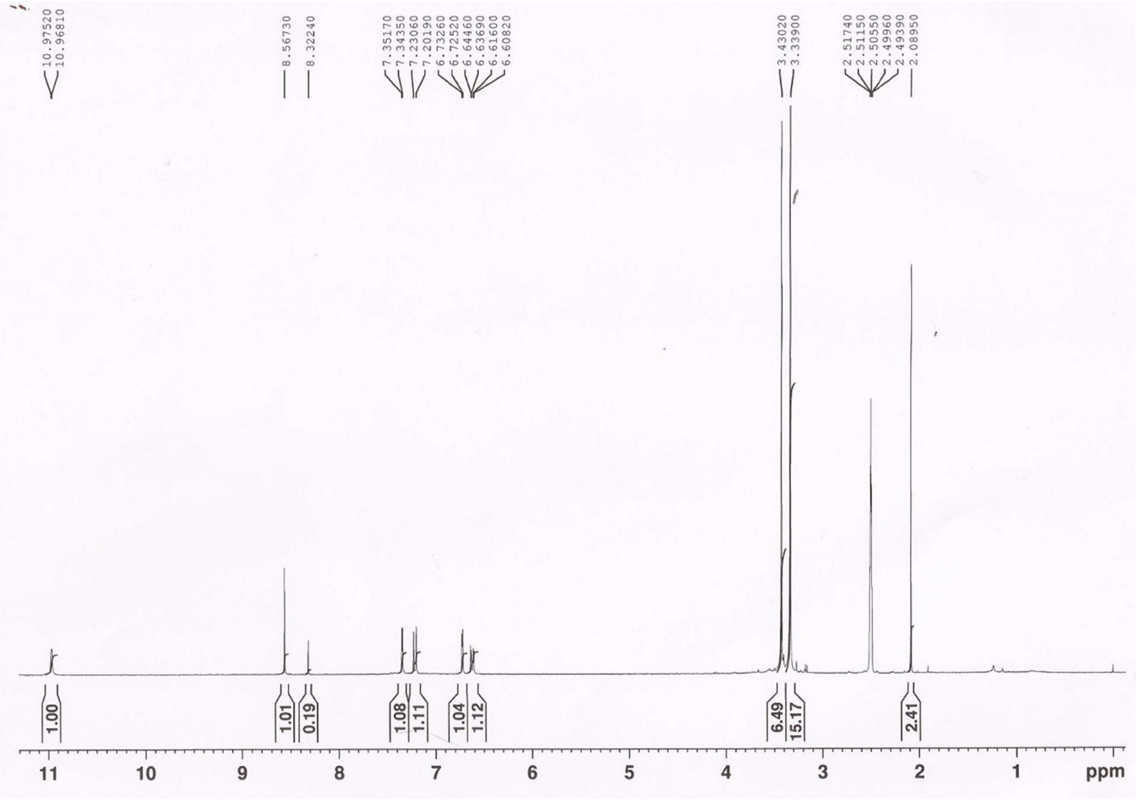

Figure S17. ^{13}C NMR spectrum kumbicin A (**2d**) (DMSO, 75.4 MHz).

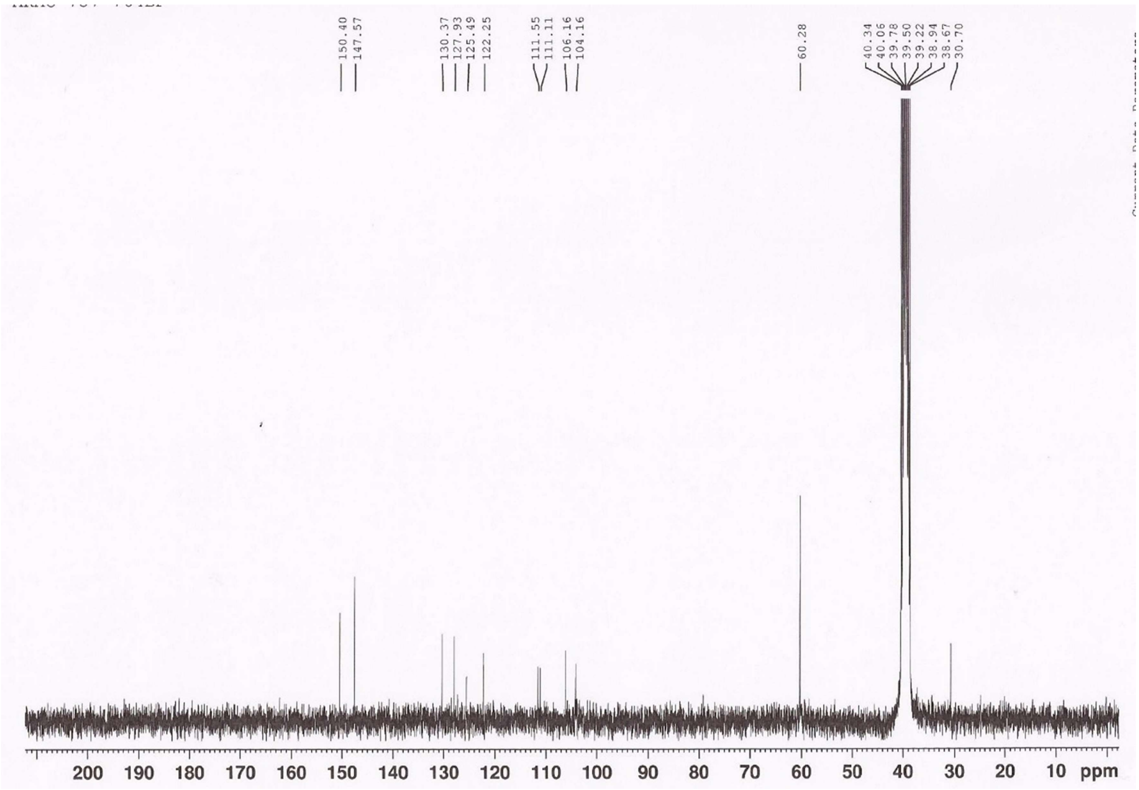

Figure S18. ^{1}H NMR spectrum of candidusin D (**2e**) (DMSO, 300.13 MHz).

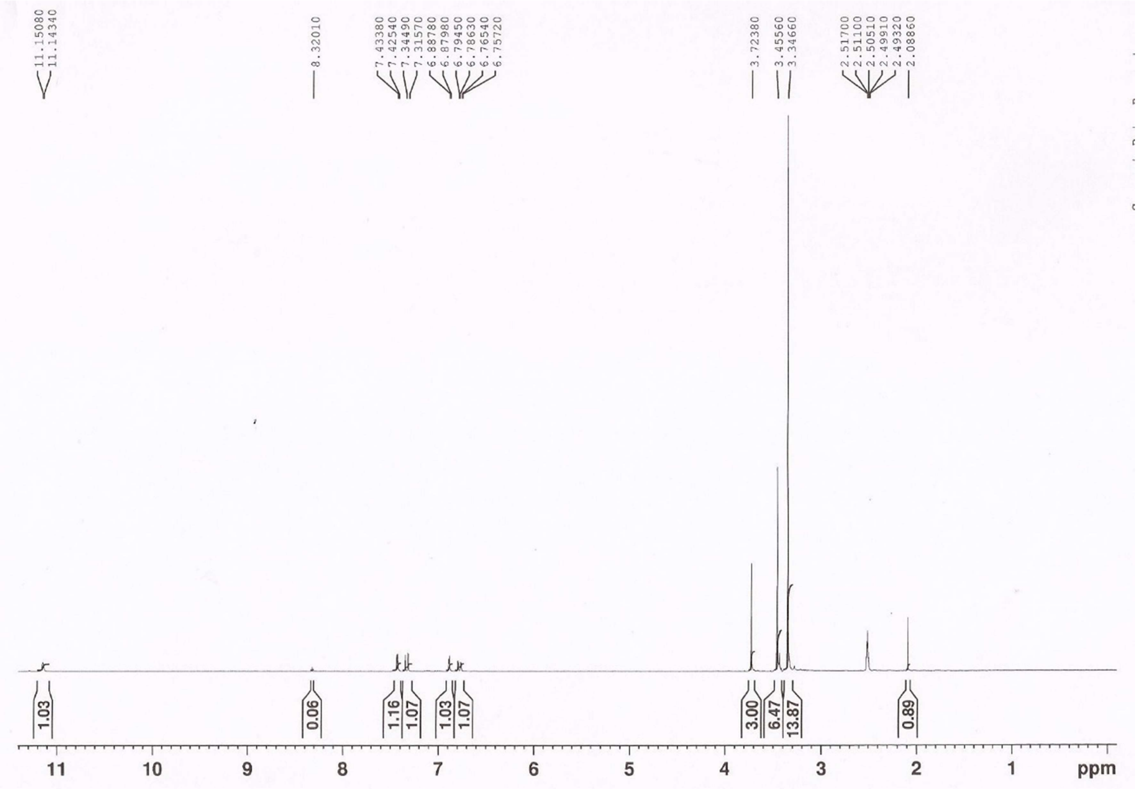

Figure S19. ^{13}C NMR spectrum of candidusin D (**2e**) (DMSO, 75.4 MHz).

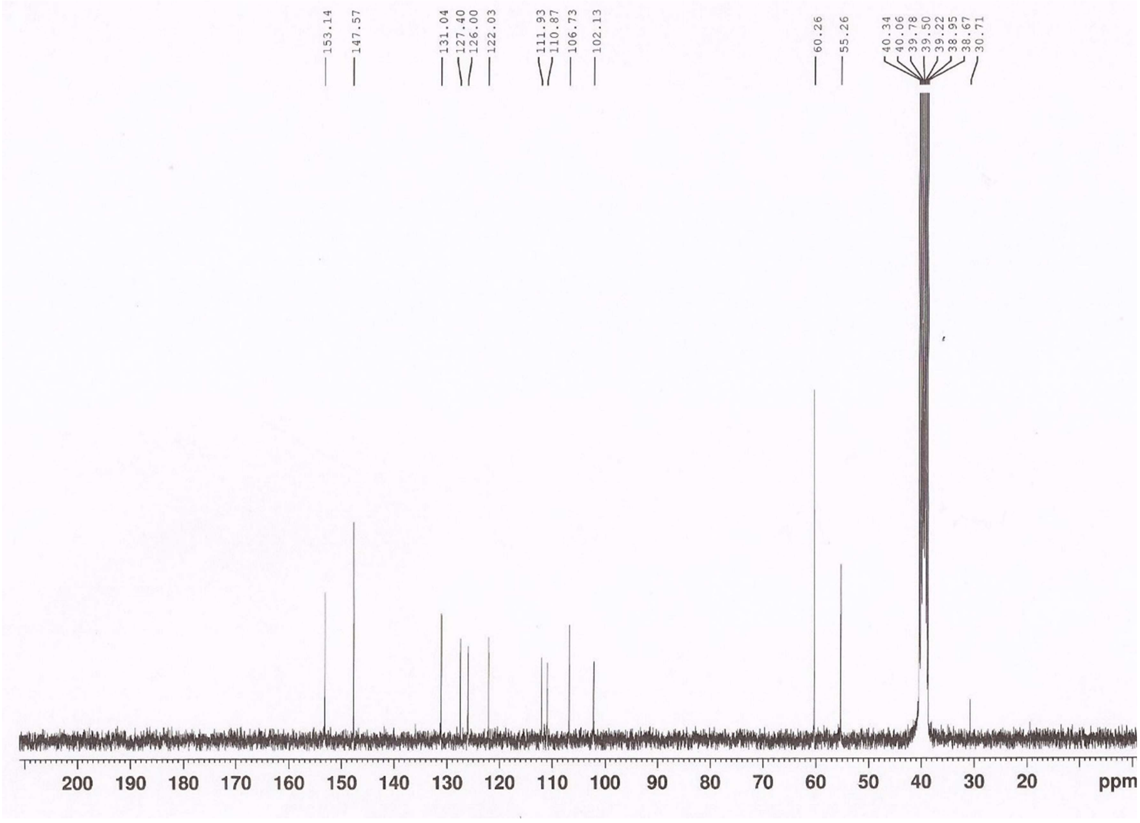

Figure S20. ^{1}H NMR spectrum of 2”-oxoasterriquinol D methyl ether (**3**) (DMSO, 300.13 MHz).

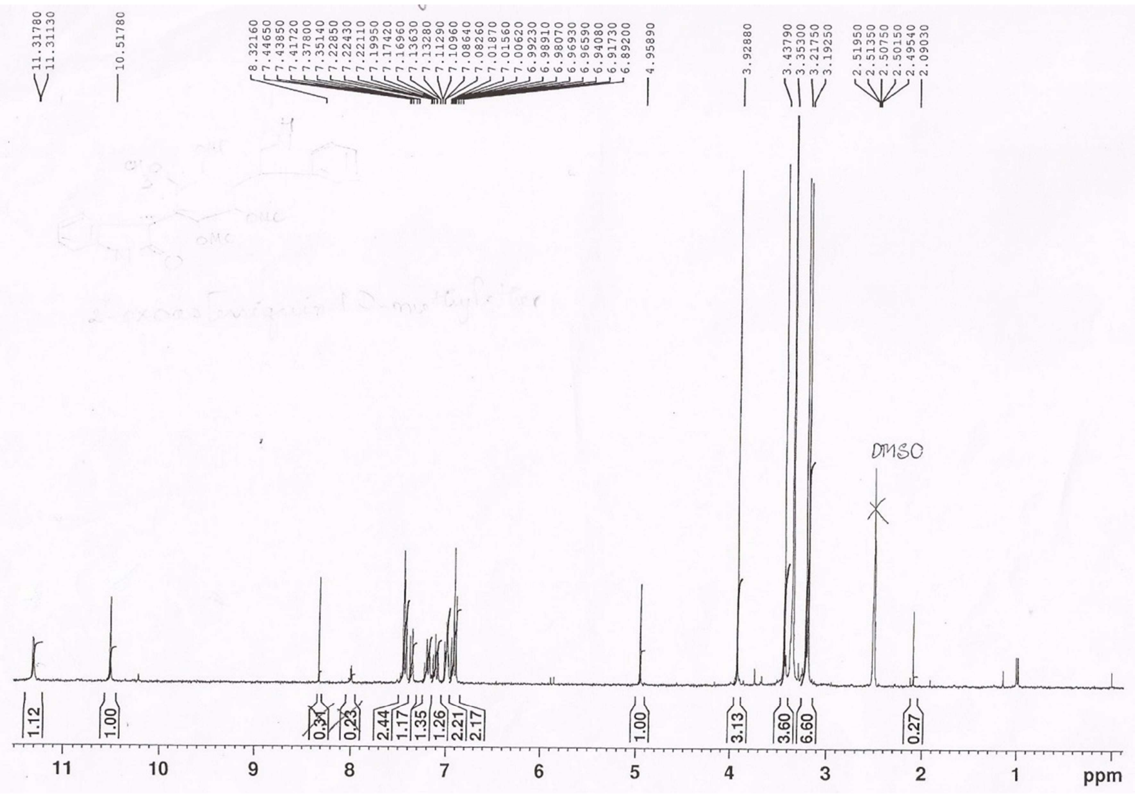

Figure S21. ^{13}C NMR spectrum of 2”-oxoasterriquinol D methyl ether (**3**) (DMSO, 75.4 MHz).

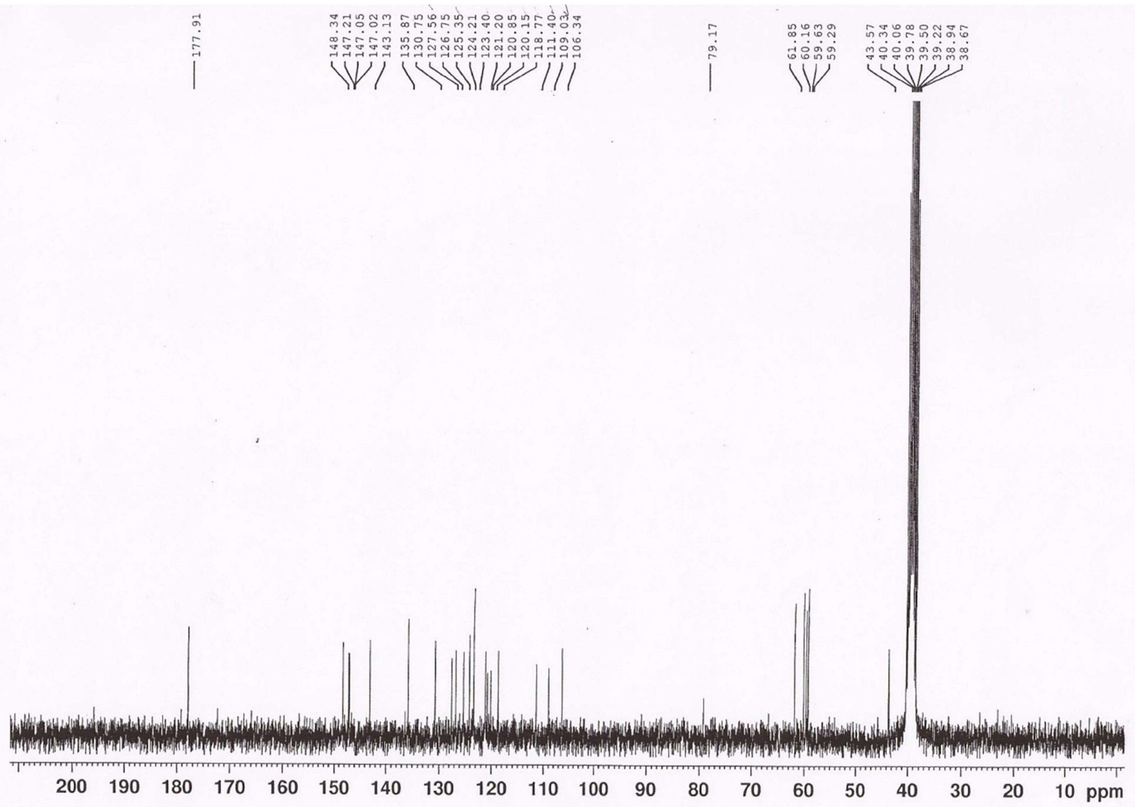

Figure S22. ^{1}H NMR spectrum of kumbicin D (**4**) (DMSO, 300.13 MHz).

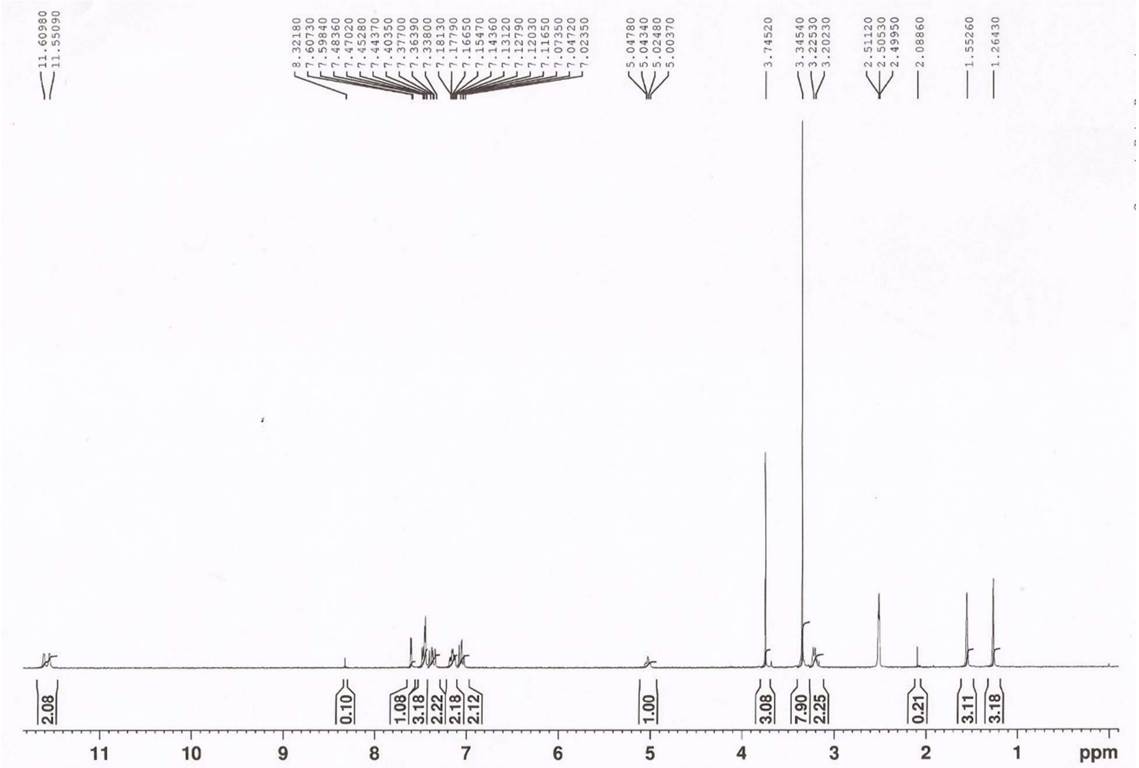

Figure S23. ^{13}C NMR spectrum of kumbicin D (**4**) (DMSO, 75.4 MHz).

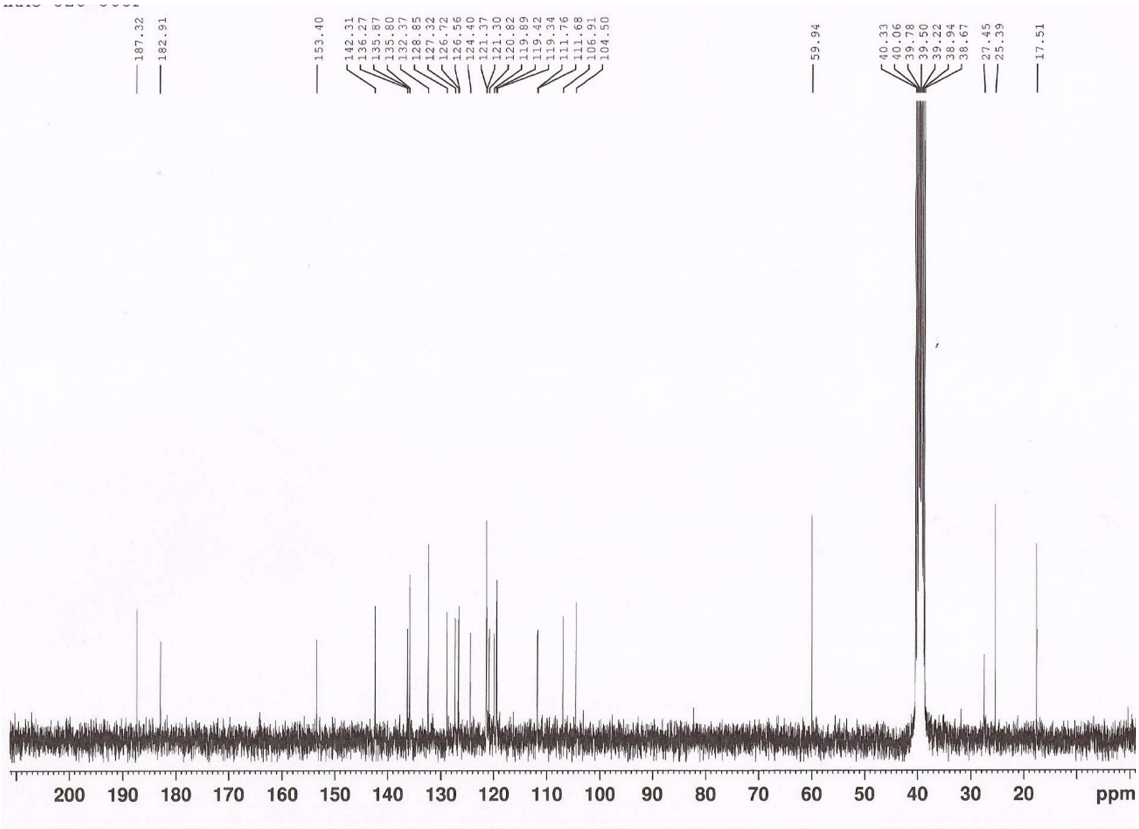

Figure S24. ^{1}H NMR spectrum of preussin (**5a**) (DMSO, 300.13 MHz).

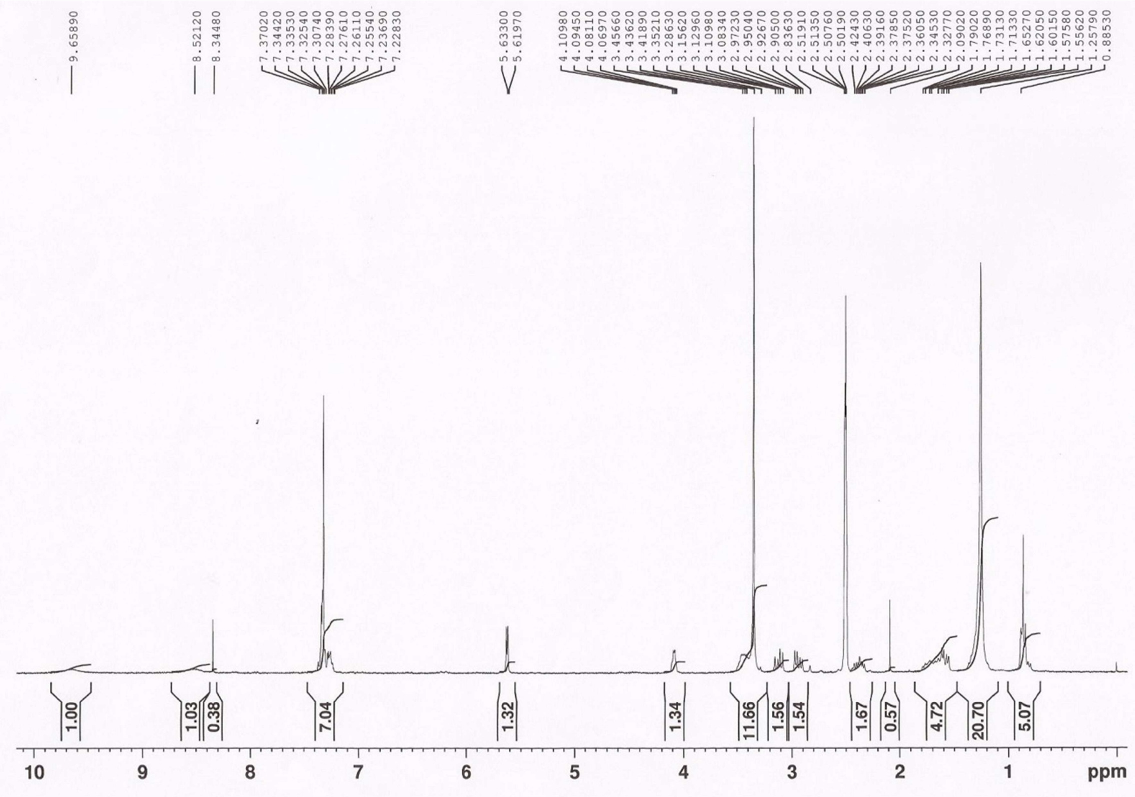

Figure S25. ^{13}C NMR spectrum of preussin (**5a**) (DMSO, 75.4 MHz).

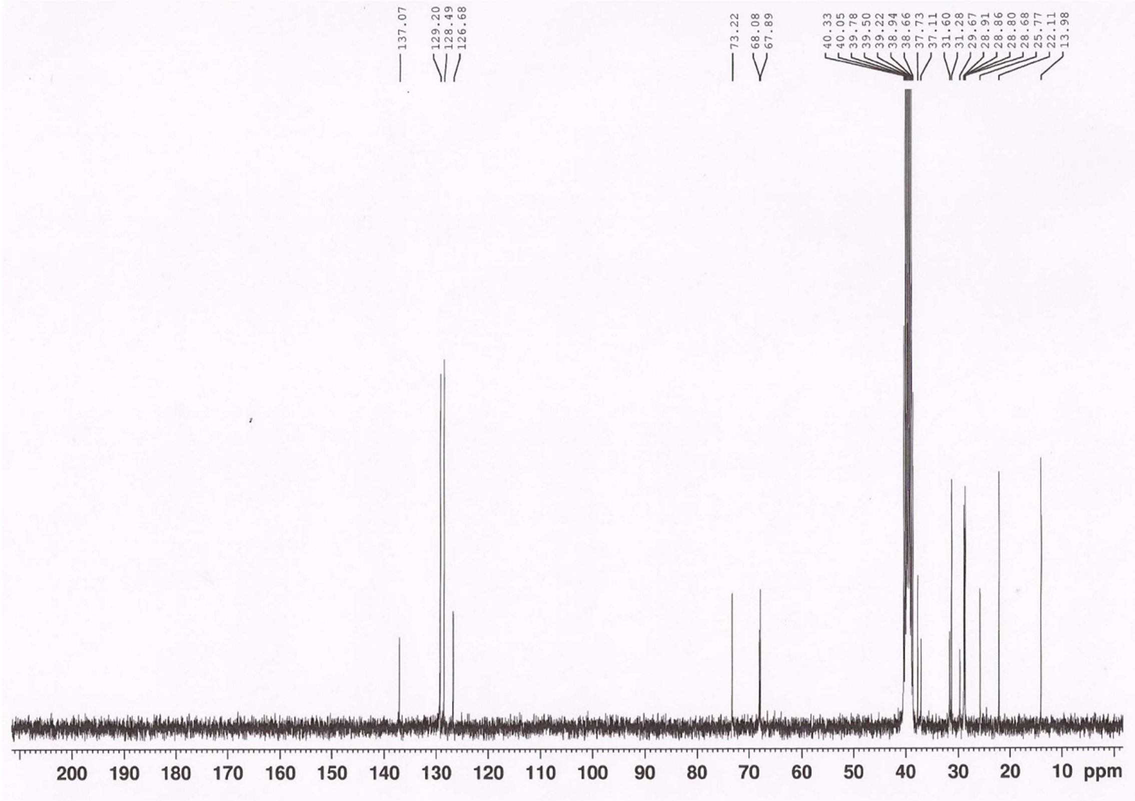

Figure S26. ^{1}H NMR spectrum of preussin C (**5b**) (DMSO, 500.13 MHz).

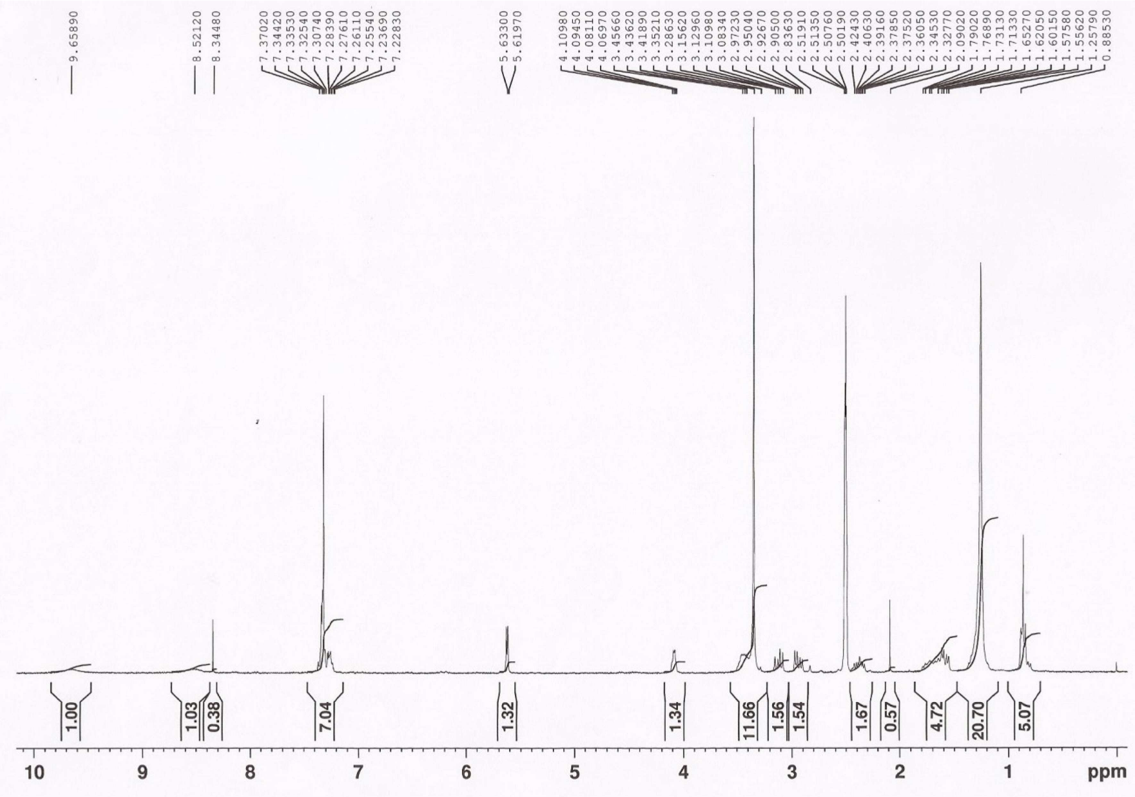

Figure S27. ^{13}C NMR spectrum of preussin C (**5b**) (DMSO, 125.4 MHz).

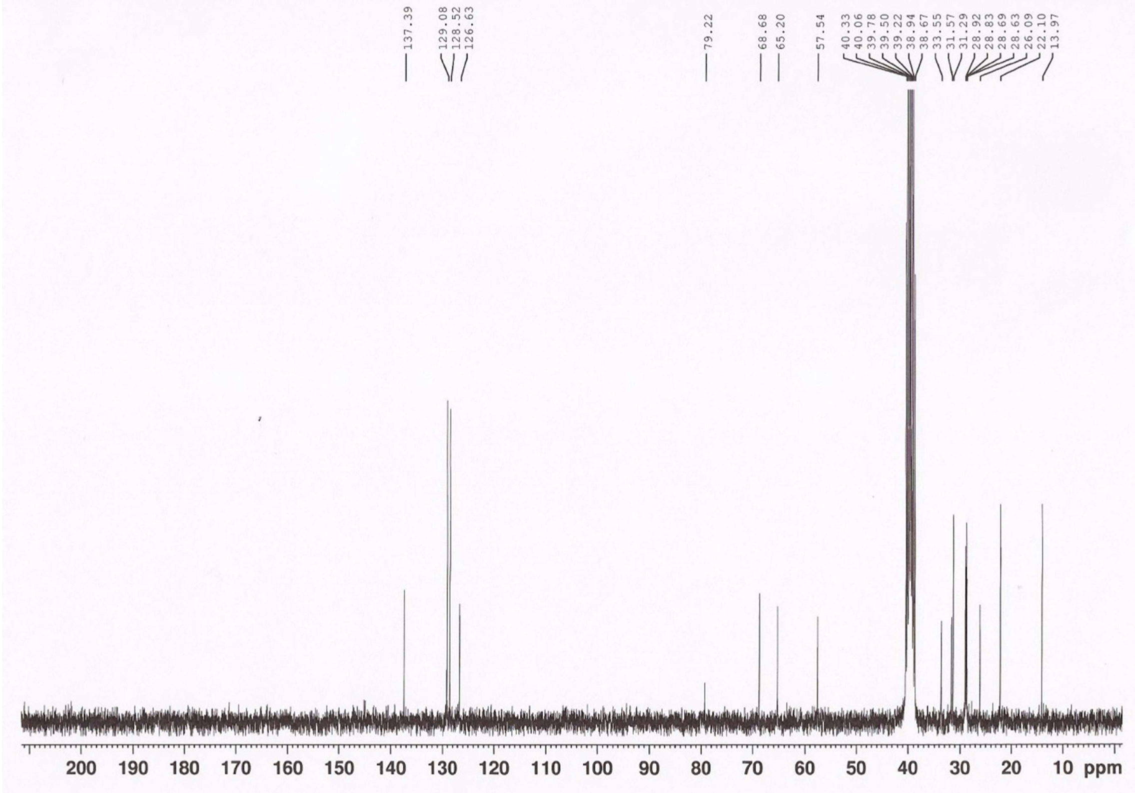

Figure S28. COSY spectrum of preussin C (**5b**) (DMSO, 500.13 MHz).

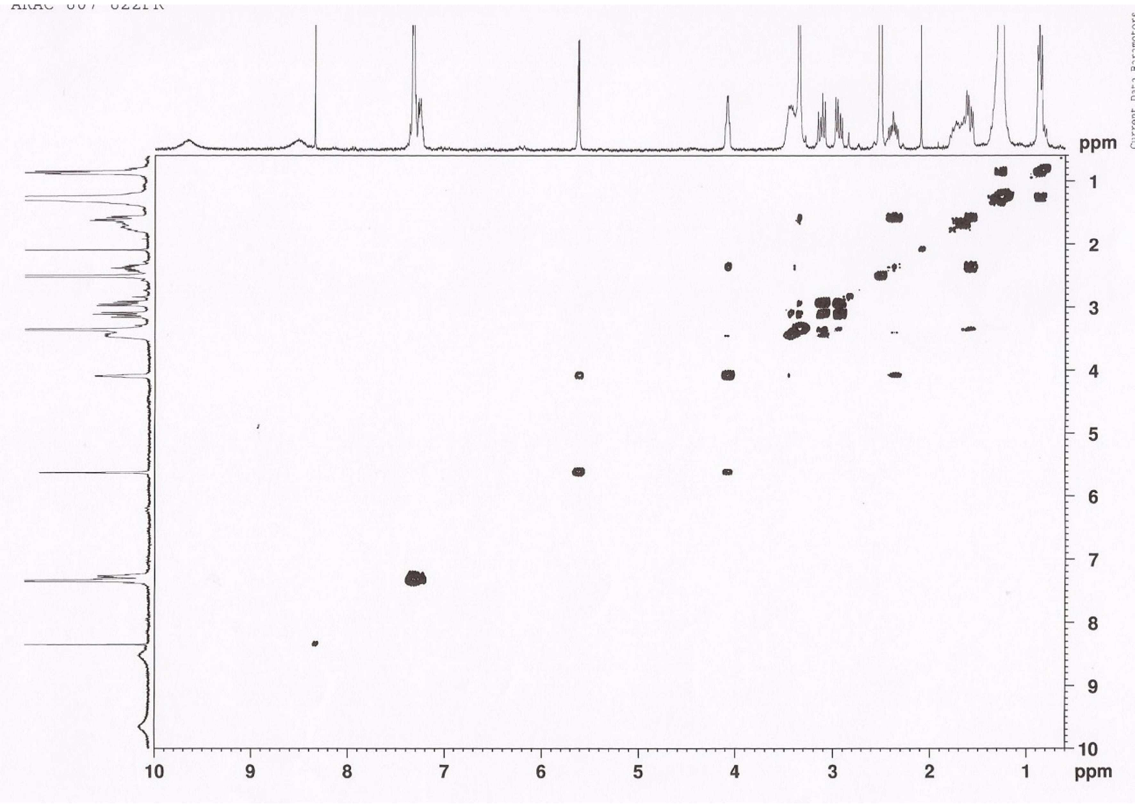

Figure S29. HSQC spectrum of preussin C (**5b**) (DMSO, 500.13 MHz).

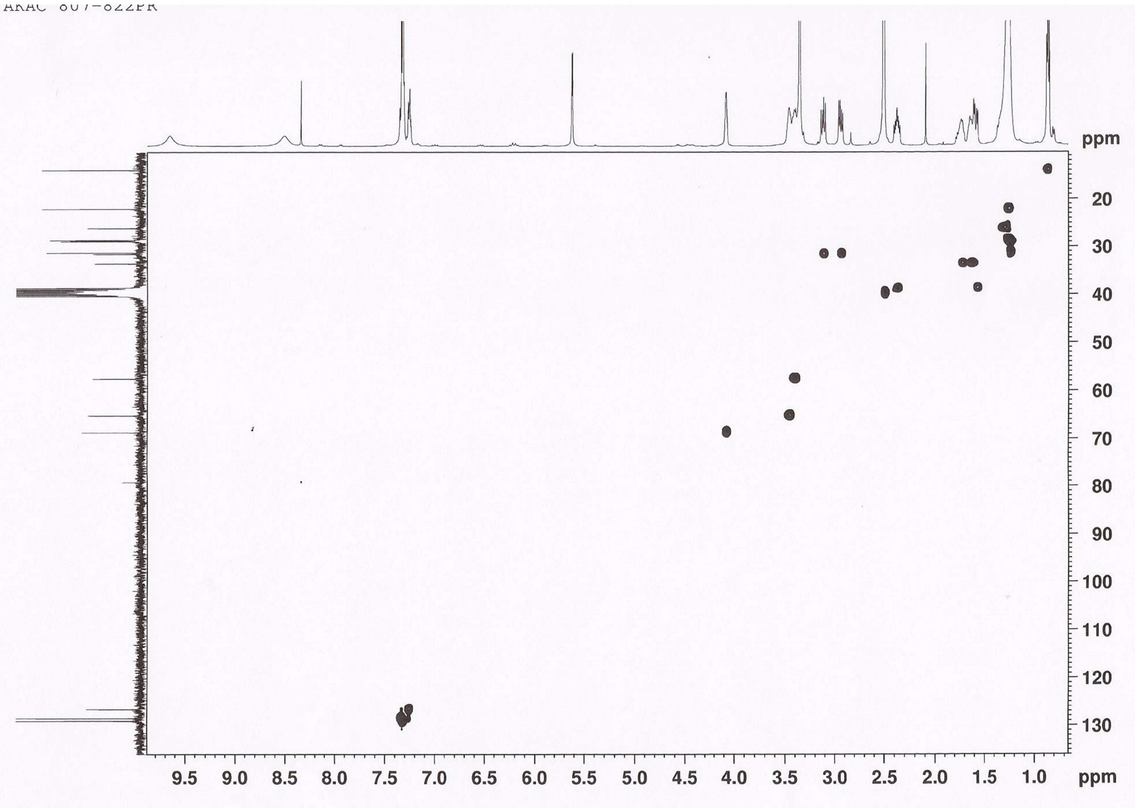

Figure S30. HMBC spectrum of preussin C (**5b**) (DMSO, 500.13 MHz).

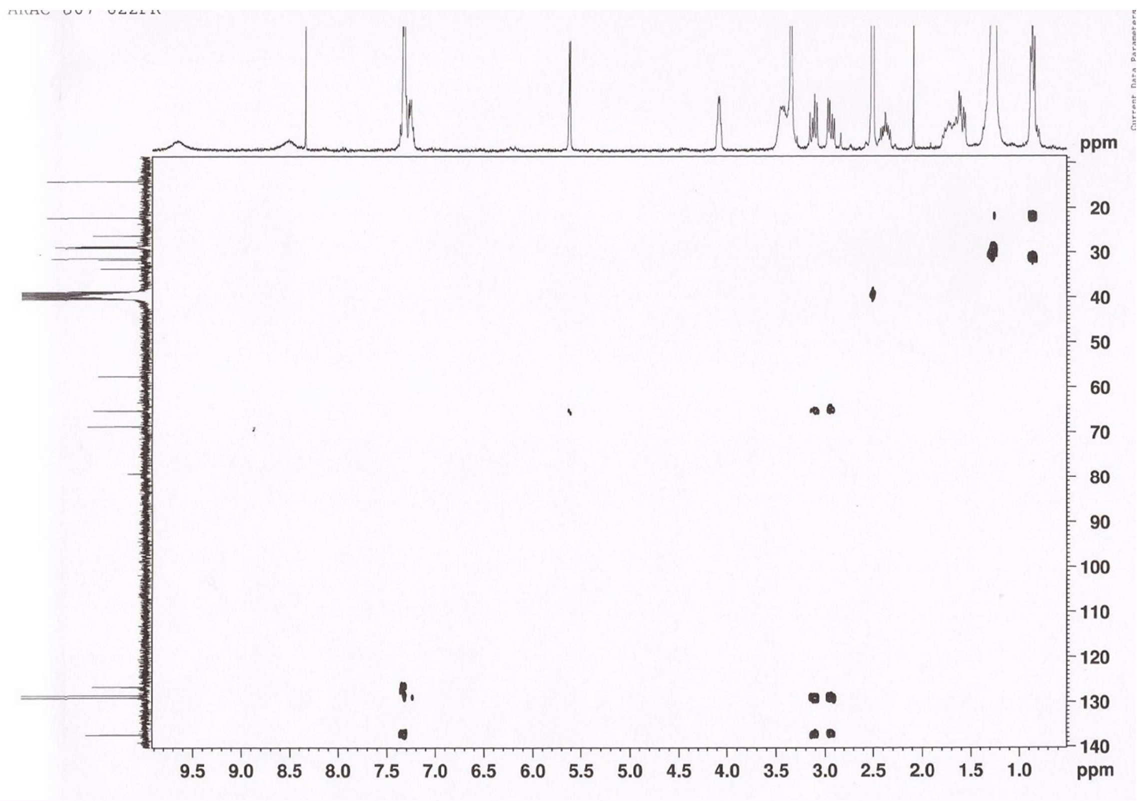

Figure S31. NOESY spectrum of preussin C (**5b**) (DMSO, 500.13 MHz).

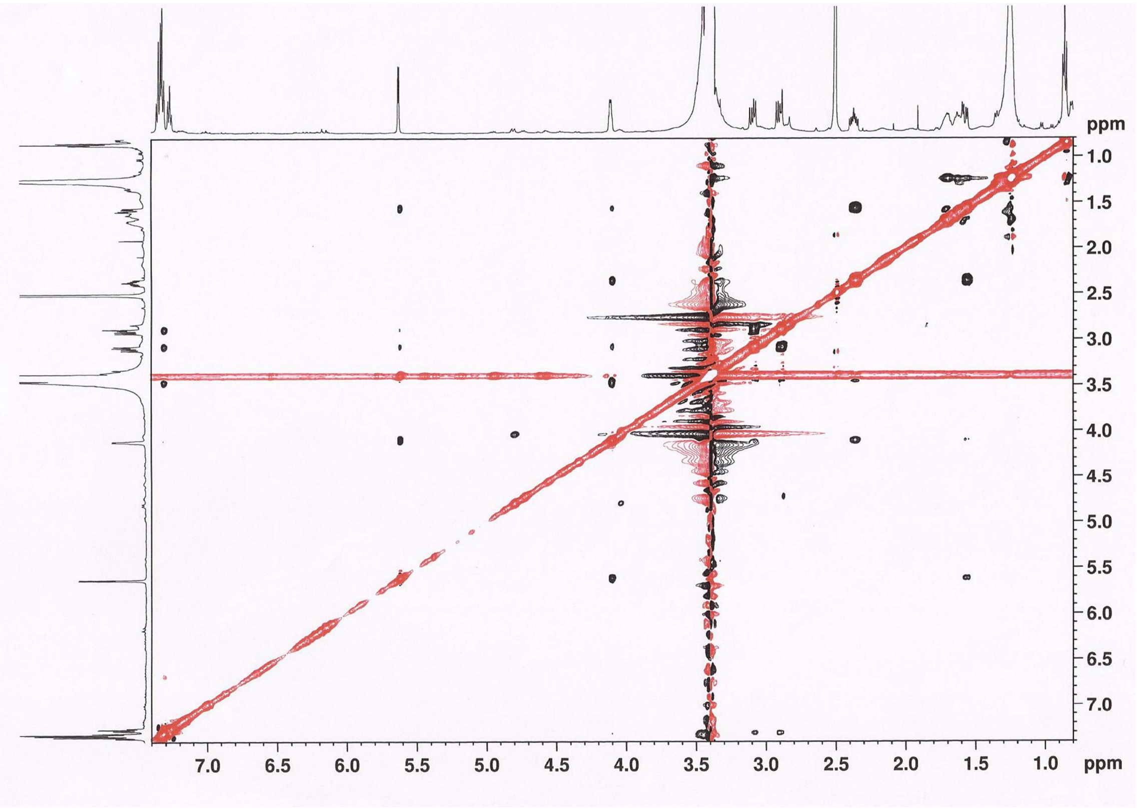

Figure S32. ^{1}H NMR spectrum of (3*S*, 6*S*)-3,6-dibenzylpiperazine-2,5-dione (**6**) (DMSO, 300.13 MHz).

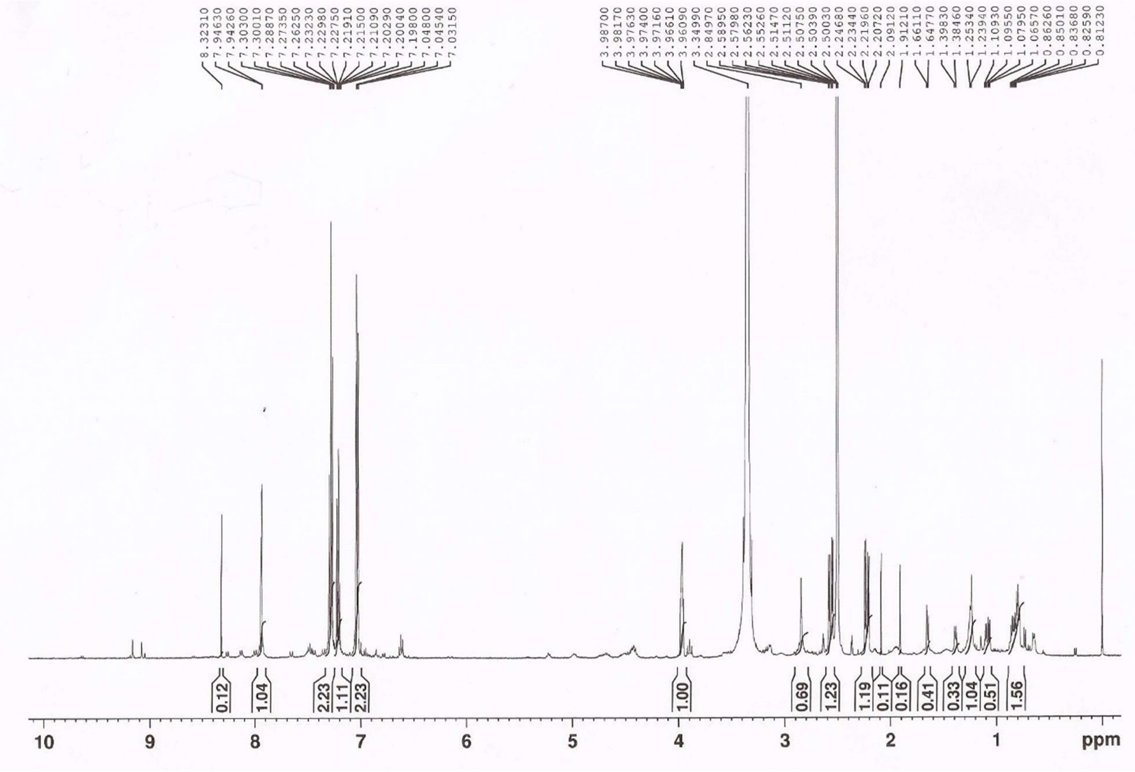

Figure S33. ^{13}H NMR spectrum of (3*S*, 6*S*)-3,6-dibenzylpiperazine-2,5-dione (**6**) (DMSO, 75.4 MHz).

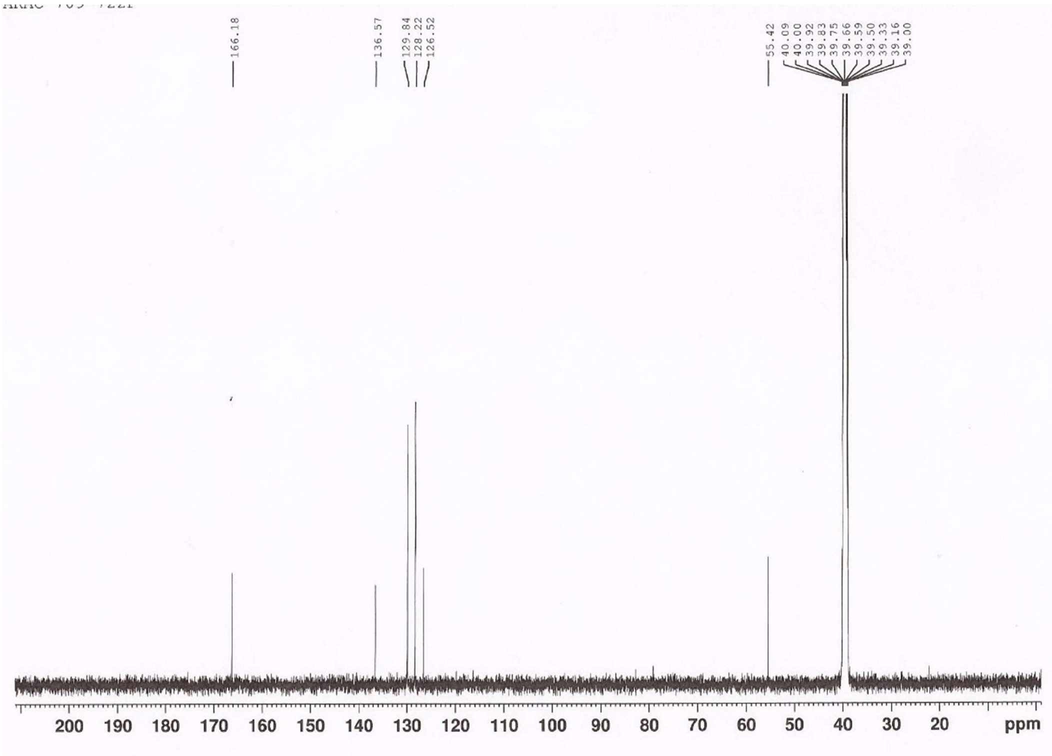

Figure S34. ^{1}H NMR spectrum of 4-(acetylamino)benzoic acid (**7**) (DMSO, 300.13 MHz).

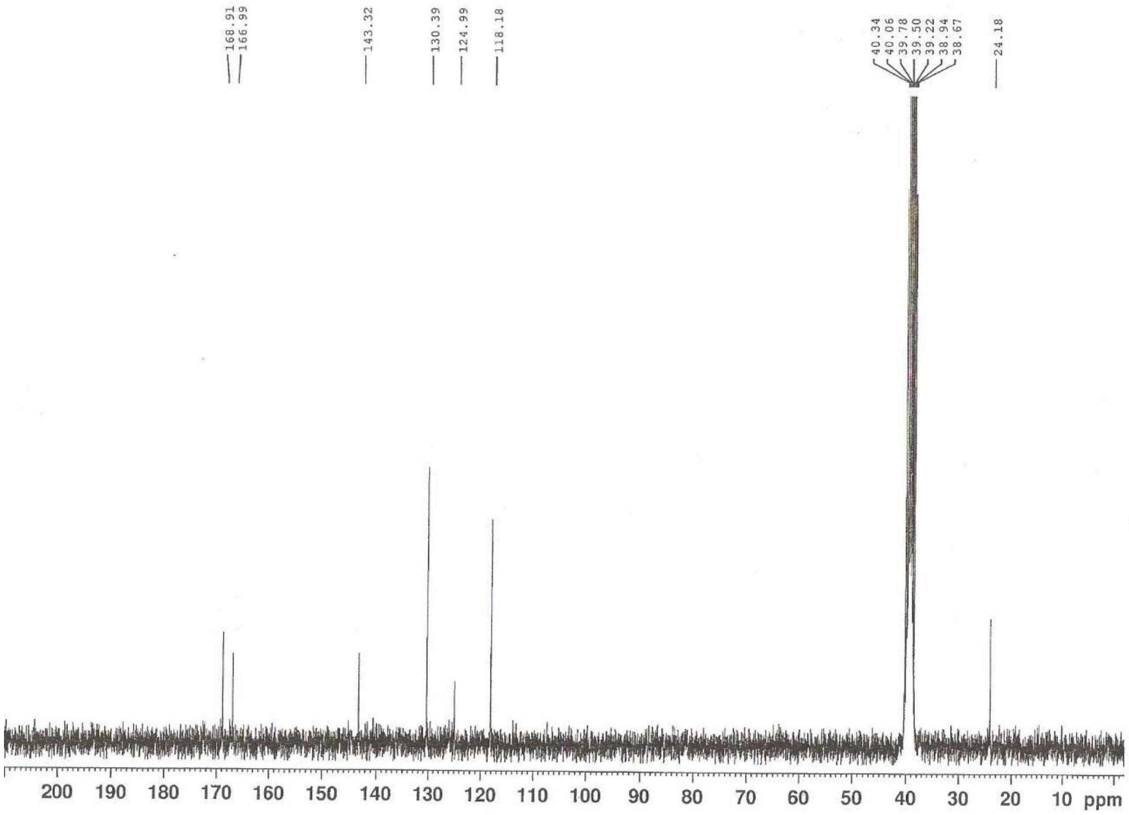

Figure S35. ^{13}C NMR spectrum of 4-(acetylamino)benzoic acid (**7**) (DMSO, 75.4 MHz).

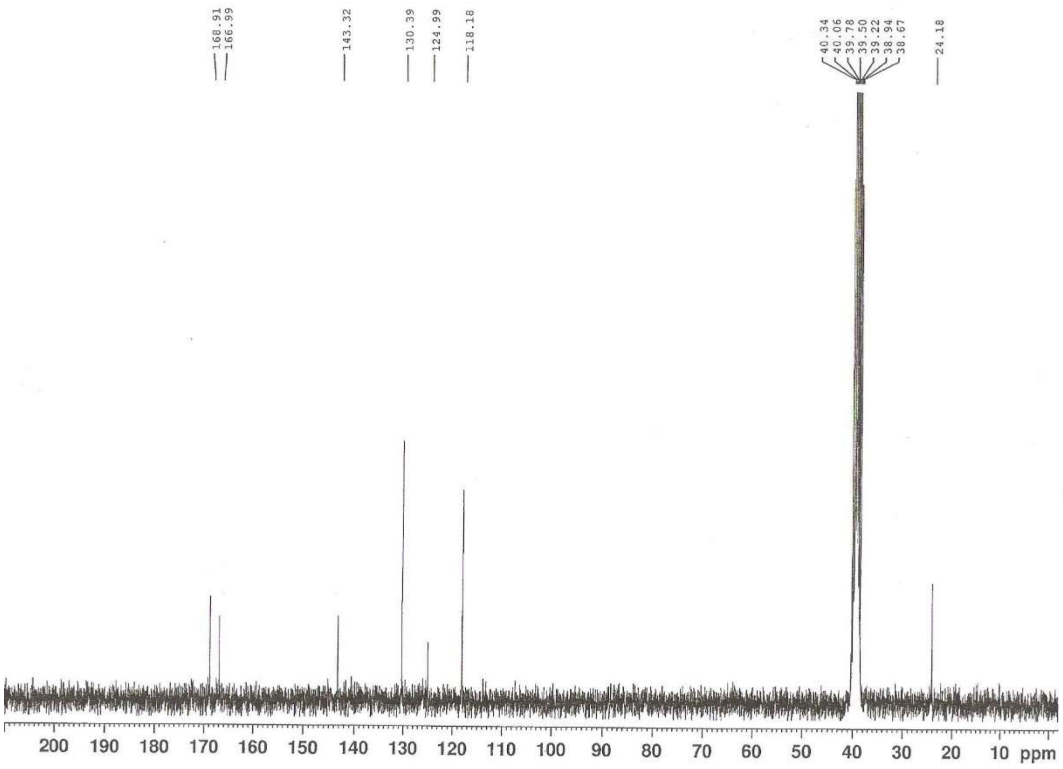

Table 1S. ^{1}H NMR (DMSO, 300.13 MHz) of **2a-d**.

δ$_H$ (*J* in Hz)				
Position	**2a**	**2b**	**2c**	**2d**
NH-1’	11.29, d (1.9)	10.98, d (2.0)	11.15, d (1.9)	10.97, d (2.1)
2’	7.47, d (2.5)	7.36, d (2.5)	7.43, d (2.5)	7.35, d (2.5)
3’	-	-	-	-
4’	7.42, d (7.5)	6.74, d (2.3)	6.88, d (2.4)	6.73, d (2.2)
5’	7.10, ddd (7.5, 7.5, 1.1)	-	-	-
6’	7.12, ddd (7.5, 7.5, 1.1)	6.63, dd (8.6, 2.3)	6.78, dd (8.7, 2.4)	6.63, dd (8.6, 2.3)
7’	7.45, d (7.5)	7.22, d (8.6)	7.33, d (8.7)	7.22, d (8.6)
NH-1”	11.29, d (1.9)	11.28, d (1.9)	11.29, d (1.9)	10.97, d (2.1)
2”	7.47, d (2.5)	7.46, d (2.5)	7.47, d (2.5)	7.35, d (2.5)
3”	-	-	-	-
4”	7.42, d (7.5)	7.41, d (7.5)	7.42, d (7.5)	6.73, d (2.2)
5”	7.01, ddd (7.5, 7.5, 1.1)	7.01, ddd (7.5, 7.5, 1.1)	7.01, ddd (7.5, 7.5, 1.1)	-
6”	7.12, ddd (7.5, 7.5. 1.1)	7.12, ddd (7.5, 7.5. 1.1)	7.12, ddd (7.5, 7.5. 1.1)	6.63, dd (8.6, 2.3)
7”	7.45, d (7.5)	7.44, d (7.5)	7.44, d (7.5)	6.73, d (2.3)
OMe-1	3.44, s	3.44, s	3.46, s	3.43, s

OMe-2	3.44, s	3.44, s	3.43, s	3.43, s
OMe-4	3.44, s	3.44, s	3.43, s	3.43, s
OMe-5	3.44, s	3.44, s	3.46, s	3.43, s
OMe-5’	-	-	3.72, s	-
OMe-5”	-	-	-	-
OH-5’	-	8.57, brs	-	8.57, brs
OH-5”	-	-	-	8.57, brs

Table 2S. ^{13}C NMR (DMSO, 75.4 MHz) of **2a-d**.

δ_H , type				
Position	**2a**	**2b**	**2c**	**2d**
1	147.6, C	147.6, C	147.6, C	147.6, C
2	147.6, C	147.6, C	147.6, C	147.6, C
3	122.2, C	121.9, C	122.0, C	122.3, C
4	147.6, C	147.6, C	147.6, C	147.6, C
5	122.2, C	147.6, C	147.6, C	147.6, C
6	147.6, C	122.5, C	122.2, C	122.3, C
2’	125.2, CH	125.5, CH	126.0, CH	125.5, CH
3’	106.9, C	106.1, C	106.7, C	106.2, C
4’	120.3, CH	104.2, CH	102.0, C	104.2, CH
5’	118.7, CH	150.4, C	153.4, C	150.4, C
6’	120.8, CH	111.1,CH	110.9, CH	111.1, CH
7’	111.4, CH	111.6, CH	111.9, CH	111.6, CH
8’	135.9, C	130.4, C	131.0, C	130.4, C
9’	127.0, C	127.9, C	127.4, C	127.9, C
2”	125.2, CH	125.2, CH	125.2, CH	125.5, CH
3”	106.9, C	107.0, C	106.9, C	106.2, C
4”	120.3, CH	120.2, CH	120.3, CH	104.2, CH
5”	118.7, CH	118.7, CH	118.7, CH	150.4, C
6”	120.8, CH	120.8, CH	120.8, CH	111.1, CH
7”	111.4, CH	111.4, CH	111.4, CH	111.6, CH
8”	135.9, C	135.9, C	135.9, C	130.4, C
9”	127.0, C	127.1, C	127.0, C	127.9, C
OMe-1	60.3, CH_3	60.3, CH_3	60.3, CH_3	60.3, CH_3
OMe-2	60.3, CH_3	60.3, CH_3	60.3, CH_3	60.3, CH_3
OMe-4	60.3, CH_3	60.3, CH_3	60.3, CH_3	60.3, CH_3
OMe-5	60.3, CH_3	60.3, CH_3	60.3, CH_3	60.3, CH_3

OMe-5’	-	-	55.2, CH_3	-
OMe-5”	-	-	-	-

Table 3S. Comparison of ^{1}H and ^{13}C NMR (DMSO, 300.13 and 75.4 MHz) of **3** with 2”-oxoasterriquinol D methyl ether ($CDCl_3$, 300.13 and 75.4 MHz).

**3**			2”-oxoasterriquinol D methyl ether [17]	
Position	δ_C , type	δ_H (*J* in Hz)	δ_C , type	δ_H (*J* in Hz)
1	148.3, C	-	148.6, C	-
2	147.2, C	-	147.4, C	-
3	124.2, C	-	123.7, C	-
4	147.1, C	-	147.5, C	-
5	147.0, C	-	147.8, C	-
6	124.2, C	-	123.5, C	-
1’	-	11.31, d (2.0)	-	8.37, brs
2’	125.4, CH	7.44, d (2.4)	124.3, CH	7.29, d (3)
3’	106.3, C	-	108.4, C	-
4’	120.2, CH	7.36, d (8.0)	121.1, CH	7.54, d (6)
5’	118.8, CH	6.99, dd (7.1, 7.5)	119.7, CH	7.11, dd (9, 6)
6’	120.9, CH	7.11, ddd (7.5, 7.5, 1.1)	121.9, CH	7.19, m
7’	111.4, CH	7.43, d (8.8)	110.9, CH	7.41, d (9)
8’	135.9, C	-	135.8, C	-
9’	126.8, C	-	127.1, C	-
1”	-	10.52, brs	-	7.81, brs
2”	177.9, CO	-	179.3, CO	-
3”	43.6, CH	4.96, s	44.0, CH	5.15, s
4”	123.4, CH	6.99, d (7.6)	124.0, CH	7.07, d (6)
5”	121.2, CH	6.92, ddd (7.1, 7.1, 1.0)	122.3, CH	6.98, dd (9, 6)
6”	127.6, CH	7.20, ddd (7.9, 7.9, 1.4)	127.7, CH	7.22, m

7”	109.0, CH	6.90, d (7.6)	109.2, CH	6.93, d (9)
8”	143.1, C	-	141.5, C	-
9”	130.8, C	-	130.9, C	-
OMe-1	61.9, CH_3	3.93,s	62.0, CH_3	4.00, s
OMe-2	59.3, CH_3	3.19, s	60.5, CH_3	3.50, s
OMe-4	59.6, CH_3	3.22, s	59.9, CH_3	3.30, s
OMe-5	60.2, CH_3	3.44, s	60.1, CH_3	3.30, s

Table 4S. ^{1}H and ^{13}C NMR data (DMSO, 300.13 and 75.4 MHz) of kumbicin D (**4**).

Position	δ_C , type	δ_H (*J* in Hz)
1	182.9, CO	-
2	153.4, C	-
3	124.4, C	-
4	187.3, CO	-
5	142.3, C	-
6	136.3, C	-
7	27.5, CH_2	3.21, d (6.9)
8	121,2, CH	5.02, t (6.3)
9	132.4, C	-
10	25.4, CH_3	1.55, s
11	17.5, CH_3	1.26, s
1’	-	11.61, brs
2’	127.3, CH	7.45, d (2.7)
3’	106.9, C	-
4’	119.9, CH	7.35, d (8.0)
5’	119.3, CH	7.05, dd (7.9, 7.9)
6’	121.3, CH	7.15, ddd (7.0, 7.0, 1.0)
7’	111.7, CH	7.46, d (8.0)

8’	135.9, C	-
9’	126.7, C	-
1”	-	11.55, brs
2”	128.9, CH	7.60, d (2.7)
3”	104.5, C	-
4”	120.8, CH	7.39, d (8.0)
5”	119.4, CH	7.05, dd (7.9, 7.9)
6”	121.4, C	7.16, ddd (7.0, 7.0, 1.0)
7”	111.8, CH	7.47, d (8.0)
8”	135.8, C	-
9”	126.6, C	-
OMe-2	59.9, CH_3	3.74, s
